# The ILLUMINATE natural history study in colony-stimulating factor 1 receptor-related adult-onset leukoencephalopathy with axonal spheroids and pigmented glia

**DOI:** 10.1093/braincomms/fcag271

**Published:** 2026-07-14

**Authors:** David S Lynch, Charles Wade, Ludger Schöls, Stefanie N Hayer, Jeffrey M Gelfand, Wolfgang Köhler, Christa-Caroline Bergner, Paulo Victor Sgobbi de Souza, Elizabeth C Finger, Nicole I Wolf, Shanice Beerepoot, Jennifer L Orthmann-Murphy, Tomasz Chmiela, Raj Rajagovindan, Donald G McLaren, Francois Gaudreault, Christian Mirescu, Denitza Raitcheva, Andreas Meier, David Gray, Juan Chavez, Petra Kaufmann, Zbigniew K Wszolek

**Affiliations:** Department of Neuromuscular Diseases, UCL Queen Square Institute of Neurology and NIHR University College London Hospitals Biomedical Research Centre, London WC1N 3BG, UK; Queen Square Multiple Sclerosis Centre, Department of Neuroinflammation, UCL Queen Square Institute of Neurology, University College London, London WC1B 5EH, UK; Department of Neurology, Hertie Institute for Clinical Brain Research and German Center of Neurodegenerative Diseases, Tübingen University Hospital, Tübingen 72076, Germany; Department of Neurology, Hertie Institute for Clinical Brain Research and German Center of Neurodegenerative Diseases, Tübingen University Hospital, Tübingen 72076, Germany; Institute of Human Genetics, University Medical Centre Hamburg-Eppendorf, Hamburg 20246, Germany; UCSF Weill Institute for Neurosciences, University of California, San Francisco, San Francisco, CA 94158, USA; Department of Neurology, University of Leipzig Medical Centre, Leipzig 04103, Germany; Department of Neurology, University of Leipzig Medical Centre, Leipzig 04103, Germany; Department of Neurology and Neurosurgery, PSEG Centro de Pesquisa Clínica, São Paulo, SP 04.038-002, Brazil; Clinical Neurological Sciences, Western University, London, ON N6A 3K7, Canada; Amsterdam Leukodystrophy Center, Department of Child Neurology, Emma Children’s Hospital, Amsterdam UMC, Vrije Universiteit Amsterdam and Amsterdam Neuroscience, Amsterdam 1081 HV, the Netherlands; Amsterdam Leukodystrophy Center, Department of Child Neurology, Emma Children’s Hospital, Amsterdam UMC, Vrije Universiteit Amsterdam and Amsterdam Neuroscience, Amsterdam 1081 HV, the Netherlands; Department of Neurology, University of Pennsylvania, Philadelphia, PA 19104, USA; Department of Neurology, Mayo Clinic Florida, Jacksonville, FL 32224, USA; Department of Neurology, Faculty of Medical Sciences, Medical University of Silesia, Katowice, Poland; Formerly Vigil Neuroscience, Inc., Watertown, MA 02472, USA; Vigil Neuroscience, Inc., Watertown, MA 02472, USA; Vigil Neuroscience, Inc., Watertown, MA 02472, USA; Vigil Neuroscience, Inc., Watertown, MA 02472, USA; Vigil Neuroscience, Inc., Watertown, MA 02472, USA; Formerly Vigil Neuroscience, Inc., Watertown, MA 02472, USA; Vigil Neuroscience, Inc., Watertown, MA 02472, USA; Vigil Neuroscience, Inc., Watertown, MA 02472, USA; Vigil Neuroscience, Inc., Watertown, MA 02472, USA; Department of Neurology, Mayo Clinic Florida, Jacksonville, FL 32224, USA

**Keywords:** *CSF1R*-related leukoencephalopathy, hereditary diffuse leukoencephalopathy with spheroids, leukodystrophy, magnetic resonance imaging, primary microgliopathy

## Abstract

Colony-stimulating factor 1 receptor-related adult-onset leukoencephalopathy with axonal spheroids and pigmented glia (CSF1R-ALSP) is a rare, fatal, autosomal-dominant neurodegenerative disorder caused by pathogenic *CSF1R* variants and characterized by progressive cognitive, neuropsychiatric, and motor dysfunction, white matter lesions on brain imaging, and white matter demyelination, swollen axons, and pigmented glial cells on pathology. Limited data regarding clinical, biofluid or radiological biomarkers of disease severity are available, and no clinical trial endpoints have yet been validated. The objectives of this first-of-its-kind, prospective, observational natural history study were to characterize the clinical trajectory of CSF1R-ALSP and to identify and evaluate key biomarkers and clinical endpoints indicative of disease severity and progression. ILLUMINATE (NCT05020743) was a multicentre, noninterventional natural history study of adults with CSF1R-ALSP and prodromal carriers of *CSF1R* variants. Participants were followed for up to 36 months, with clinical assessments, fluid biomarkers and volumetric MRI assessments of brain atrophy collected at screening and every 6 months. This study was terminated early (4 June 2025). The analyses reported here include data collected through 19 February 2025. Of 53 participants, 19 were prodromal and 34 were symptomatic (11 of whom had a history of haematopoietic stem cell transplant and 23 who did not). Mean participant age was 47.8 (standard deviation, 4.5) years, and 36.4% were female. Prodromal participants remained relatively stable over 36 months, with little change in neurological function, neurodegeneration biomarkers or radiological disease burden. Impaired neurological function, MRI characteristics of CSF1R-ALSP, and elevated NfL (neurofilament light chain; neurodegeneration biomarker) and GFAP (glial fibrillary acidic protein; astrogliosis biomarker) levels were more pronounced at baseline and often showed progression over time among symptomatic participants who had not previously received haematopoietic stem cell transplant compared with participants who had previously received haematopoietic stem cell transplant. Significant correlations were observed at baseline and longitudinally between MRI measures of brain atrophy and clinical outcome measures. Based on the fluid biomarkers, MRI measures, and clinical outcome assessments evaluated here, active neurodegeneration, widespread changes visualized on brain MRI, and impaired cognitive and motor function were observed in symptomatic patients with CSF1R-ALSP. The neurological impairment can be assessed using the Montreal Cognitive Assessment and Cortical Basal ganglia Functional Scale. Our data suggest that quantification of brain atrophy using MRI volumetry is a potential biomarker of disease severity and progression in CSF1R-ALSP. It is hoped that this report will contribute to the understanding of disease progression in CSF1R-ALSP and inform future drug development.

## Teaser

Lynch *et al.* report an observational study to chart the course of colony-stimulating factor 1 receptor-related adult-onset leukoencephalopathy with axonal spheroids and pigmented glia, a rare leukoencephalopathy, showing neurological impairment, widespread brain MRI changes, and high neurodegeneration markers in symptomatic patients. Results increase disease understanding and inform future drug development.

## Graphical Abstract

**Graphical Abstract:**
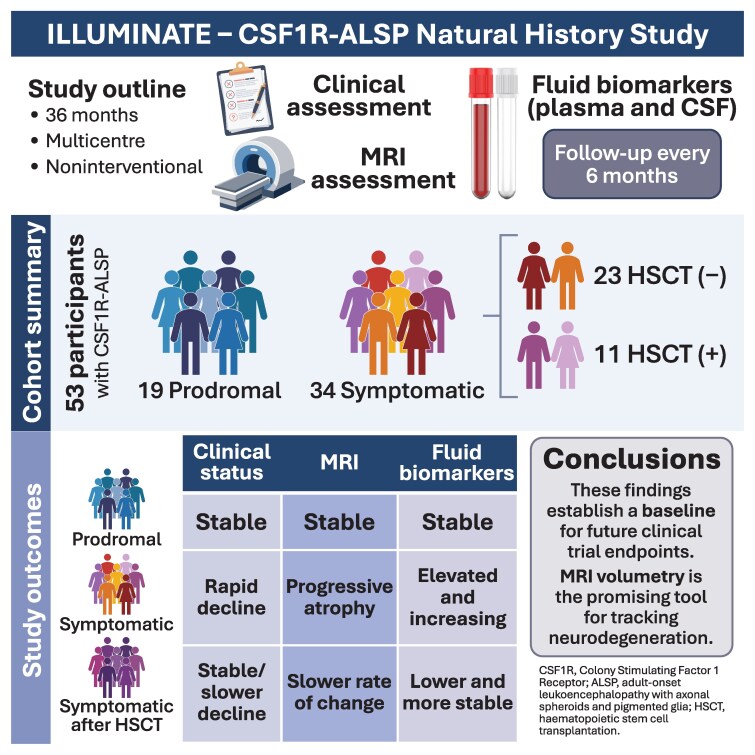

## Introduction

Colony-stimulating factor 1 receptor-related adult-onset leukoencephalopathy with axonal spheroids and pigmented glia (CSF1R-ALSP) is a rare, autosomal dominant and progressive fatal leukoencephalopathy.^1-3^ It is caused by pathogenic *CSF1R* gene variants,^1-3^ which were recently estimated to occur with a frequency of 281 per million individuals.^4^ CSF1R-ALSP is characterized pathologically by brain white matter demyelination, microglia loss, swollen axons and pigmented glial cells.^1,5^ Brain magnetic resonance imaging (MRI) shows early white matter lesions followed by brain atrophy.^1,5^ Neurologic examination shows progressive cognitive decline and other cortical disturbances (e.g. aphasia, agraphia, apraxia), neuropsychiatric symptoms (e.g. behavioural and/or personality changes), motor dysfunction symptoms (e.g. both pyramidal and extrapyramidal), speech disturbance, and bulbar and cerebellar signs.^1,6,7^ Symptom onset frequently occurs in the early 40s [mean (range) age, 43 (18‒86) years].^8^

CSF1R-ALSP signs and symptoms substantially impact patients’ activities of daily living, including mobility, executive functioning, mood/behaviour, self-care and maintaining employment.^1,6^ In later stages, individuals progress to loss of speech and voluntary movement and become confined to bed; death is most often due to infections such as pneumonia.^6^ CSF1R-ALSP progresses rapidly, with progression from symptom onset to incapacitation within 3‒4 years and death within 6‒8 years.^1,7^

Initial misdiagnosis of CSF1R-ALSP is common and occurs in ∼50% of patients,^8^ resulting in delays in clinical care initiation, symptomatic treatment and genetic counselling, as well as missed opportunities for clinical trial participation.^8,9^ Introduction of the ICD-10 code (G93.44) in October 2023^10^ and increasing accessibility of genetic testing^11^ may shorten the diagnostic timeline, potentially mitigating the currently prolonged diagnostic odyssey for people with CSF1R-ALSP.

Brain MRI can help in CSF1R-ALSP diagnosis and assessing disease severity and progression.^1,2,6,7,12,13^ In a study involving 15 patients with CSF1R-ALSP, a semiquantitative, clinician-assessed severity (Sundal) scoring system was proposed for diagnostic evaluation and to track disease progression.^12^ Additionally, results showed a distinctive pattern of bilateral, asymmetric white matter lesions with prefrontal predominance and brain atrophy, primarily in the cortical and central regions, with increased ventricle volume. Longitudinal MRI scans show disease progression and severity,^13^ particularly volumetric MRI measures (e.g. lesion volume, ventricle volume), which can be used to track disease progression.^14^ Furthermore, positive association was observed between progressive white matter degeneration and brain atrophy on MRI with increasing functional disability, as assessed via modified Rankin scale, and increasing disease duration in a cohort of eight patients with CSF1R-ALSP.^15^ Thus, longitudinal white matter lesion tracking and volumetric measurements on brain MRI patterns may be used for evaluating therapeutic interventions for CSF1R-ALSP.^1,14,16^

No approved pharmacotherapies are yet available that can modify CSF1R-ALSP disease course.^17^ Haematopoietic stem cell transplantation (HSCT) is the only potentially disease-modifying option but is available and appropriate for only a subset of patients; furthermore, data on its efficacy in CSF1R-ALSP have yielded inconsistent results.^18-22^ HSCT efficacy in CSF1R-ALSP has not been evaluated in controlled clinical trials^1^; however, stabilization of motor function, cognitive outcomes and MRI lesions were reported following HSCT in a small series of patients.^18-20,22-24^ Similarly, in a recent study, HSCT was found to replace CSF1R-deficient microglia in four individuals with CSF1R-ALSP, halting disease progression, with no exacerbation in brain atrophy and stabilization of motor and cognitive function.^25^ Although long-term treatment outcomes from these case studies were limited and post-HSCT disease course varied,^18-20,23,24^ a retrospective study of 15 patients who had undergone HSCT suggests that patients with milder cognitive impairment, predominant motor presentation and younger age before HSCT had relatively more favourable outcomes.^21^ Another retrospective study of 17 patients found that a reduced-intensity conditioning regimen may display similar neurological outcomes to myeloablative transplantation.^22^ The widespread HSCT applicability for patients with CSF1R-ALSP is currently limited by risks of complications (e.g. chemotherapy conditioning-associated toxicity, infections resulting from immunosuppression, graft-versus-host disease, transient worsening of symptoms, increased treatment-related mortality),^19,21,26^ disease severity at diagnosis and access to appropriate transplant donors. To provide an alternative, a phase 2 clinical trial was conducted in parallel with the natural history study reported here to evaluate iluzanebart, a monoclonal antibody TREM2 agonist, as a potential treatment for CSF1R-ALSP. Although iluzanebart demonstrated a favourable safety, tolerability and pharmacokinetic profile, no beneficial effects on biomarker or clinical efficacy endpoints were observed.^27^

**Figure 1:**
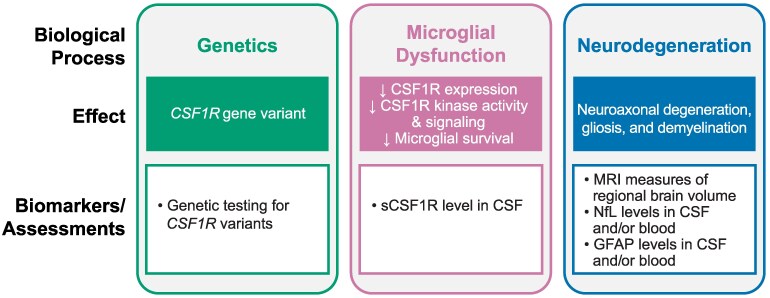
**Biological processes, their effect, and biomarkers/assessments.** CSF, cerebrospinal fluid; CSF1R, colony-stimulating factor 1 receptor; GFAP, glial fibrillary acidic protein; MRI, magnetic resonance imaging; NfL, neurofilament light chain; sCSF1R, soluble colony-stimulating factor 1 receptor.

Thus, a significant unmet need remains for disease-modifying treatment for CSF1R-ALSP to reverse, delay or stop disease progression.^1,5,6^ Since the 2011 report of the genetic and aetiological basis of CSF1R-ALSP,^2^ limited data have been published on biomarkers of disease biology and severity, and no clinical endpoints have yet been established that measure the range of neurological manifestations of CSF1R-ALSP. The investigators, therefore, considered data from clinical research in other neurological diseases with characteristics similar to CSF1R-ALSP.^28,29^ Of interest were biomarkers that would link the underlying CSF1R-ALSP (i.e. a pathogenic *CSF1R* gene variant) to the purported primary disease mechanism of microglial dysfunction by targeting the cascade of resulting neurodegeneration (Fig. 1). The aim of this first prospective, observational study was to better understand the natural history of CSF1R-ALSP and develop and evaluate potential key markers of disease progression in patients with CSF1R-ALSP and asymptomatic carriers of *CSF1R* gene variants.

## Materials and methods

### Study design

**Figure 2:**
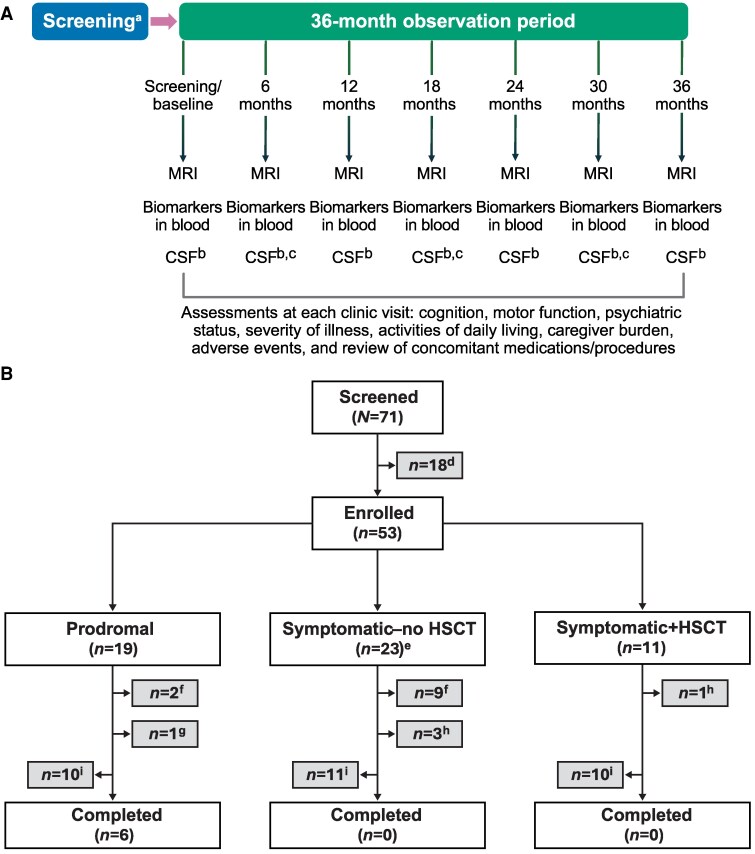
**ILLUMINATE (A) study design and (B) participant disposition.**  ^a^Screening/baseline assessments could be completed at more than 1 study visit; however, all assessments were completed within 28 days. ^b^Optional substudy. ^c^Collected at study site in Brazil only. ^d^Screen failure. ^e^Does not include 3 additional participants who enrolled after the 19 February 2025, data cut-off date. ^f^Discontinued to enrol in phase 2 IGNITE clinical study. ^g^Discontinued due to lost to follow-up. ^h^Discontinued due to withdrawal by subject. ^i^Were active in study as of 19 February 2025, data cut-off date. CSF, cerebrospinal fluid; HSCT, haematopoietic stem cell transplant; MRI, magnetic resonance imaging.

ILLUMINATE was a noninterventional, prospective, multicentre, observational study (ClinicalTrials.gov identifier: NCT05020743) of patients with definitive CSF1R-ALSP and those with prodromal CSF1R-ALSP with a documented *CSF1R* gene variant. The study was conducted at 12 sites in Brazil, Canada, Germany, the Netherlands, the United Kingdom and the United States^30^ between September 2021 and June 2025. Eligible participants entered the observation period for up to 36 months, with study visits to assess disease status at screening/baseline and every 6 months thereafter (Fig. 2A). The study included an optional substudy conducted at select sites to assess cerebrospinal fluid (CSF) biomarkers at screening/baseline, 12, 24 and 36 months (all sites) and additionally at month 6 (Brazil only). The study was terminated on 4 June 2025, by the sponsor after a lack of efficacy was demonstrated in the sponsor’s IGNITE phase 2 CSF1R-ALSP interventional study (NCT05677659).^27^ Therefore, ILLUMINATE data through 19 February 2025 were analysed and are reported here.

This study was conducted in accordance with consensus ethical principles derived from international guidelines, including the Declaration of Helsinki and Council for International Organizations of Medical Sciences International Ethical Guidelines, International Council for Harmonization guidelines for Good Clinical Practice, and all applicable laws and regulations. The study protocol and all related documents were reviewed and approved by the institutional review board or independent ethics committee at each participating site (Supplementary Table 1). All participants or their legally authorized representative (with subject assent) provided written informed consent before participation.

### Participants

All participants must be ≥18 years of age at screening, have a documented *CSF1R* variant and have brain MRI findings consistent with CSF1R-ALSP (i.e. bilateral cerebral white matter lesions). Participants with definitive (symptomatic) CSF1R-ALSP who were ambulatory with or without aids must have demonstrated clinical progression of CSF1R-ALSP within the past year, a baseline Montreal Cognitive Assessment Scale (MoCA) score of ≥12, and more than two clinical sign or symptom findings in any of the following categories: cognitive impairment or psychiatric problem, pyramidal signs on neurological examination, extrapyramidal features (e.g. rigidity, tremor, abnormal gait and bradykinesia) or epilepsy. Participants with prodromal CSF1R-ALSP must have none or two or fewer CSF1R-ALSP-related clinical signs or symptoms. Participants were excluded if they had any other neurological or psychiatric disease associated with cognitive, motor or behavioural impairment similar to CSF1R-ALSP. Participants who had previously undergone HSCT were enrolled if they met screening criteria.

Participants who met the criteria for symptomatic CSF1R-ALSP were required to have a designated study partner (i.e. caregiver) who was able to spend ≥4 h per week with them, able and willing to assist the participant in complying with study requirements, provide information during study visits and sign a study partner informed consent form. Participants with prodromal CSF1R-ALSP who later progressed during the study to definitive CSF1R-ALSP were also required to have a designated study partner for subsequent visits.

### Endpoints and assessments

#### Fluid biomarkers

Serum for measurement of neurofilament light chain (NfL) and glial fibrillary acidic protein (GFAP) levels was collected at baseline and months 6, 12, 18, 24, 30 and 36/end of treatment (ET). Blood samples were collected in Vacutainer^®^ SST^TM^ gold-cap tubes containing clotting activators (Becton, Dickinson and Company, Franklin Lakes, NJ, USA) and were allowed to clot at room temperature and centrifuged at 1000*×g* for 15 min. Extracted serum was stored in aliquots of up to 0.5 ml at −80°C.

CSF was collected for measurement of NfL, GFAP, soluble TREM2 (sTREM2), soluble CSF1R (sCSF1R) and osteopontin/secreted phosphoprotein 1 (OPN/SPP1) levels at baseline and months 12, 24 and 36/ET; for the study site in Brazil, CSF was also collected at month 6. Lumbar punctures were performed at the same time of day across visits, from which 10 ml of CSF was collected and divided into 0.5-ml aliquots and stored at less than −70°C, avoiding freeze–thaw cycles.

Detailed methods for quantification of fluid biomarkers are in Supplementary Methods and Supplementary Table 2.

#### MRI assessments and processing

Volumetric and structural brain MRI measurements were conducted at baseline and at months 6, 12, 18, 24, 30 and 36/ET. MRI scans included 3D T1-weighted and T2-weighted fluid-attenuated inversion recovery sequences. Image acquisition guidelines were established to standardize MRI acquisition across participating sites. Objective volumetric measurements of different brain regions (ventricle, total grey matter, whole brain volume, corpus callosum thickness, corpus callosum volume and white matter lesions) were conducted by trained, independent expert neuroimaging data analysts, blinded to participant-specific data, including clinical status (prodromal or definitive) and prior treatment status (non-HSCT or HSCT), using a semi-automated image analysis pipeline (details are provided in Supplementary Methods).

Sundal MRI severity score^12^ was assessed by two independent, board-certified neuroradiologists who were blinded to participant-specific data, imaging time points, clinical status, prior treatment status and any other information that could affect the blinding protocol validity. Derived from visual assessment of an MRI scan for signal intensity abnormalities across 34 brain areas, the Sundal score (range, 0‒57 points) quantifies the degree and distribution of white matter lesions, atrophy, and abnormal T2 hyperintensities using a point system (e.g. 0 = none, 1 = mild, 2 = marked), with greater disease burden indicated by higher scores (mild, 1.0‒6.0; moderate, 7.0‒15.0; severe, 16.0‒57.0).^12^ If scores from the two neuroradiologists differed by ≥5 points, a consensus meeting was held to agree on the new scoring; otherwise, the two scores were averaged.

#### Clinical assessments

Cognitive and functional assessments were conducted by trained raters at local sites at screening/baseline and months 6, 12, 18, 24, 30 and 36/ET using the MoCA and the Clinical Dementia Rating Scale plus National Alzheimer’s Coordinating Center-Frontotemporal Lobar Degeneration (CDR^®^+NACC-FTLD). The CDR + NACC-FTLD administration could be omitted at the investigator’s discretion if no signs or symptoms of cognitive impairment (i.e. normal MoCA score) were present.

MoCA, a clinician-administered, 30-point assessment, can be completed in ≈10 min and was developed as a screening tool to detect mild cognitive impairment.^31,32^ It can be used to evaluate multiple cognitive domains, including executive function, memory, visuospatial ability, language and attention, with scores ≤25 indicating cognitive impairment.^31,32^ If the participant had ≤12 years of education and a MoCA score <30, one point was added to their score.

The CDR + NACC-FTLD is a clinician-administered, global assessment tool developed by tailoring the established CDR dementia staging instrument used in patients with Alzheimer’s disease to evaluate patients with frontotemporal lobar degeneration (FTLD).^33^ Given some overlap in symptoms and signs between CSF1R-ALSP and FTLD, the CDR + NACC-FTLD was included in this study, although prior experience with the instrument was lacking in CSF1R-ALSP. The CDR + NACC-FTLD is based on a semi-structured interview with the patient and a reliable informant/caregiver to measure the severity of dementia symptoms in the following domains: memory, orientation, judgement and problem-solving, community affairs engagement, home and hobbies, behaviour, language and personal care.^33^ The first seven domains are rated on a 5-point scale (0 = normal, 0.5 = questionably or minimally impaired, 1 = mildly but definitely impaired, 2 = moderately impaired, 3 = most severely impaired); the personal care domain has no rating of 0.5 and is therefore rated on a 4-point scale. The total of the eight individual domain ratings is calculated to create the CDR + NACC-FTLD sum of boxes (CDR + NACC-FTLD-SB), with scores ranging from 0 to 24. The CDR + NACC-FTLD global score is calculated from the eight individual domains and rated on the same 5-point scale, with scores of 0‒3 and lower scores indicating better function.

Detailed methods for additional clinical assessments [Two-Minute Walk Test (2MWT)^34^; Timed Up and Go (TUG) Test^35^; Functional Assessment Questionnaire (FAQ)^36^; and Zarit Burden Interview (ZBI)^37^] are in the Supplementary Methods.

The Clinical Global Impression–Severity of Illness (CGI-S) and Change (CGI-C) and Patient Global Impression–Severity of Illness (PGI-S) and Change (PGI-C) scales were used to assess baseline disease severity and change from baseline in participants’ overall condition.^38^ For the CGI-S, clinicians rated baseline severity using a 7-point scale relative to past experience with CSF1R-ALSP patients. For the PGI-S, patients rated the current severity of their disease on a 4-point scale (1 = normal, 2 = mild, 3 = moderate, 4 = severe). For CGI-C and PGI-C, the clinician and patient, respectively, rated the participant’s overall condition on a 7-point scale that varied from 1 = very much improved to 7 = very much worse. CGI-S and PGI-S were completed at screening/baseline, and CGI-C and PGI-C were completed at months 6, 12, 18, 24, 30 and 36/ET.

The Cortical Basal ganglia Functional Scale (CBFS) evaluates experiences in daily living (EDLs), behavioural, language and cognitive impairments, and assesses functional disability in individuals with four-repeat tauopathies (e.g. corticobasal degeneration and progressive supranuclear palsy).^39^ The performance of this tool in CSF1R-ALSP has not been described in the literature. In this study, the CBFS was assessed at screening/baseline and months 6, 12, 18, 24, 30 and 36/ET. The CBFS comprises 14 questions on motor EDLs and 17 questions on nonmotor EDLs, each rated on a 5-point Likert scale ranging from 0 = normal or no problem to 4 = severe problems, with a total score of 0‒124. The questions pertain to the patients but should be answered cooperatively by the patient and caregiver, based on usual or average function over the past 2 weeks.

#### Other assessments

Demographic characteristics, details of the family/participant history of CSF1R-ALSP, and any medications taken from 30 days before screening/baseline through the last study visit were recorded. Complete physical and neurological examinations were conducted at screening/baseline and months 6, 12, 18, 24, 30 and 36/ET. Because this was a noninterventional study, the only safety assessments performed were collection of medical history, physical and neurological examinations, and pregnancy testing for women of childbearing potential.

### Statistical analysis

Analysis included all participants with available data at screening/baseline. Demographic, baseline characteristics, and clinical and biomarker assessments were summarized using descriptive statistics. Continuous variables were described using means and standard deviations (SDs) or medians and ranges where appropriate; categorical variables were described using frequencies and percentages.

Early study termination and missing data limited the feasibility of several preplanned analyses. Therefore, the statistical analysis focused on baseline data and a subset of longitudinal observations only. For outcomes for which change from baseline could be calculated (MRI ventricle volume, MoCA total score, CBFS total score), least squares (LS) means and standard errors (SEs) for each subgroup and LS mean differences from the prodromal subgroup were obtained from a mixed model for repeated measures assessing change from baseline as the dependent variable and included fixed effects of categorical subgroup, categorical visit, continuous baseline score, subgroup × visit interaction, and baseline score × visit interaction. The within-participant covariance was modelled using a compound symmetric with heterogeneous variance structure. To examine whether brain volume loss and serum fluid biomarkers correlated with clinical outcomes, Pearson correlation analyses were conducted between MRI volume measures/serum fluid biomarkers and cognitive/clinical outcome measures. Statistical analyses were performed using SAS® (SAS Institute Inc., Cary, NC, USA) version 9.4. Statistical significance was defined as *P* < 0.05, unless stated otherwise for multiplicity adjustment.

## Results

### Study population

Of the 53 enrolled participants, 19 (35.8%) were categorized at baseline as prodromal, 23 (43.4%) as symptomatic-no HSCT and 11 (20.8%) as symptomatic + HSCT (Fig. 2B). Only six participants, all in the prodromal subgroup, completed the full duration of the study by the data cut-off date of 19 February 2025. Sixteen participants discontinued the study early: 11 participants did so to enrol in the phase 2 IGNITE interventional study, 4 participants voluntarily withdrew, and 1 participant was lost to follow-up. No participant died during the study, and the remaining 31 participants were active in the study as of the 19 February 2025, data cut-off date.

Among the 53 study participants, mean ± SD age at screening was 45.4 ± 12.5 years, 58.5% were female and most were White (88.7%) (Table 1). Disease duration since diagnosis was greatest in the symptomatic + HSCT subgroup, with a median (range) of 4.1 (1.9‒5.9) years, compared with 0.4 (0.0‒5.0) years in the symptomatic-no HSCT subgroup and 0.4 (0.0‒3.0) years in the prodromal subgroup. For the 11 participants in the symptomatic + HSCT subgroup, the mean ± SD (range) time from HSCT to study entry was 2.6 ± 1.0 (1.4‒5.1) years. Detailed information on the rationale, timing and protocol used for HSCT treatment was not available as the therapy was performed outside of the study’s observation period.

**Table 1:** Baseline participant demographics and disease characteristics

Characteristic	Prodromal (*n* = 19)	Symptomatic- no HSCT (*n* = 23)	Symptomatic +HSCT (*n* = 11)
**Age at screening, years**			
Mean (SD)	45.3 (16.6)	44.5 (11.5)	47.5 (4.6)
Median (min, max)	39.0 (19.0, 76.0)	41.0 (24.0, 63.0)	49.0 (40.0, 54.0)
**Age at diagnosis,^a^ years**			
Mean (SD)	45.0 (15.0)	43.6 (11.2)	43.9 (4.6)
Median (min, max)	40.2 (24.8, 75.0)	40.6 (24.6, 63.4)	44.8 (36.8, 51.0)
**Disease duration,^a,b^ years**			
Mean (SD)	1.1 (1.3)	1.0 (1.4)	4.1 (1.5)
Median (min, max)	0.4 (0.0, 3.0)	0.4 (0.0, 5.0)	4.1 (1.9, 5.9)
**Sex, *n* (%)**			
Female	12 (63.2)	15 (65.2)	4 (36.4)
Male	7 (36.8)	8 (34.8)	7 (63.6)
**Race,^c^ *n* (%)**			
Black	1 (5.3)	3 (13.0)	0
Multiple	0	1 (4.3)	0
Unknown	0	0	1 (9.1)
White	18 (94.7)	19 (82.6)	10 (90.9)
**Ethnicity, *n* (%)**			
Hispanic or Latino	2 (10.5)	6 (26.1)	1 (9.1)
Not Hispanic or Latino	17 (89.5)	16 (69.6)	10 (90.9)
Not stated	0	1 (4.3)	0
**BMI,^d^ kg/m^2^**			
Mean (SD)	25.0 (6.1)	26.6 (3.7)	25.3 (3.7)
Median (min, max)	24.9 (13.4, 38.0)	27.4 (20.6, 33.2)	25.7 (17.7, 30.0)
**MRI measures, mean (SD)**			
Ventricle volume, ml	30.0 (15.8)	67.4 (27.0)	117.5 (38.5)
Total grey matter volume, ml	582.1 (67.0)	528.3 (55.1)	491.7 (48.0)
Corpus callosum volume, ml	31.6 (3.4)	25.8 (5.9)	19.7 (5.5)
Corpus callosum thickness, mm	6.5 (0.7)	5.0 (1.0)	3.6 (0.7)
Whole brain volume, ml	1146.9 (124.0)	1054.0 (113.7)	1016.6 (88.7)
White matter lesion volume, ml	9.8 (9.5)	33.1 (23.4)	42.9 (17.1)
Sundal MRI severity score	4.0 (4.4)	18.4 (8.9)	26.3 (6.0)
**Clinical outcome assessments, mean (SD)**			
MoCA total score^e^	27.4 (1.9)	19.7 (6.1)	18.7 (5.9)
CDR + NACC-FTLD global score	0.2 (0.3)	1.5 (0.9)	2.2 (0.8)
CDR + NACC-FTLD sum of boxes score	0.2 (0.3)	8.8 (6.4)	12.0 (5.4)
CBFS total score	3.4 (3.8)	31.2 (22.2)	35.1 (19.5)
2MWT	182.1 (127.7)	138.7 (165.4)	87.0 (51.5)
TUG	9.5 (3.5)	24.2 (29.9)	27.7 (22.2)
FAQ	0.0 (0.0)	19.8 (6.1)	18.0 (9.3)
ZBI	6.7 (4.9)	26.1 (17.2)	40.7 (25.8)

^a^Prodromal, *n* = 10; symptomatic-no HSCT, *n* = 22; symptomatic + HSCT, *n* = 11.

^b^Calculated as (date of screening − date of diagnosis)/365.25 and rounded to 1 decimal place.

^c^More than 1 race may have been selected; participants with multiple races are summarized in the ‘Multiple’ category.

^d^Prodromal, *n* = 18; symptomatic-no HSCT, *n* = 23; symptomatic + HSCT, *n* = 10.

^e^An early version of the study protocol allowed participants with MoCA scores <12 to enrol with sponsor approval, which resulted in the enrolment of 2 participants in the symptomatic-no HSCT subgroup.

2MWT, 2-Minute Walk Test; BMI, body-mass index; CBFS, Cortical Basal ganglia Functional Scale; CDR + NACC-FTLD, Clinical Dementia Rating Scale plus National Alzheimer’s Coordinating Center-Frontotemporal Lobar Degeneration; FAQ, Functional Activities Questionnaire; HSCT, haematopoietic stem cell transplant; MoCA, Montreal Cognitive Assessment; MRI, magnetic resonance imaging; SD, standard deviation; TUG, Timed Up and Go; ZBI, Zarit Burden Interview.

*CSFIR* variants identified in the study and their classification and evidence summary are presented in Supplementary Fig. 1 and Table 3*. CSF1R* variants in most participants [50/53 (94.3%)] were within the tyrosine kinase domains (roughly mapping to exons 13‒15 and 17‒21) and were missense [42/53 (79.2%)]. Of the 37 *CSF1R* variants identified in 53 participants, only eight were identified in more than one participant: p.I794T (c.2381T > C) in seven participants; p.A781_N783del (c.2342_2350del) in three participants; and p.R579W (c.1735C > T), p.G589E (c.1766G > A), p.Q642X (c.1924C > T), p.R782H (c.2345G > A), c.2442 + 1G > A, p.G798R, p.S836N (c.2507G > A) and p.Y886SfsX56 (c.2656_2657insC) in two participants each. Some participants were related to each other.

### Biomarker assessments

**Figure 3:**
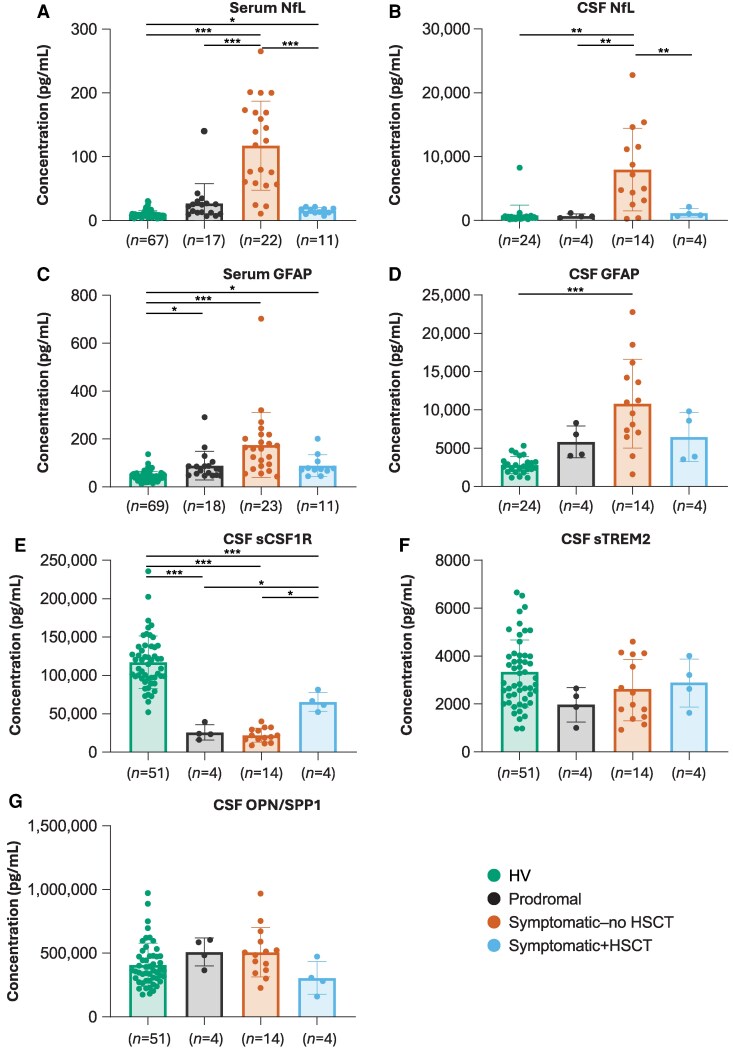
**Fluid biomarker levels in ILLUMINATE patients at baseline versus HVs from iluzanebart phase 1 study.** Bars and error bars represent mean and SD, respectively, and circles represent individual patient values. Fluid biomarker levels in healthy individuals are from the first-in-human single-/multiple-ascending dose study of iluzanebart in HVs (Supplementary Table 4).^40^ Pairwise *post hoc* comparisons were conducted using two-sample *t*-tests with Welch’s correction to account for unequal variances. Adjustment for multiple comparisons within each analyte was performed using the Dunnett T3 procedure. Only statistically significant comparisons are shown here; all *P* values are reported in the Supplementary Table 6. *P < 0.05; **P < 0.01; ***P < 0.001. One HV had a CSF NfL concentration that was 4.5 SDs higher than the mean; a sensitivity analysis omitting this individual resulted in mean (SD) of 477 (255) pg/ml. CSF, cerebrospinal fluid; GFAP, glial fibrillary acidic protein; HSCT, haematopoietic stem cell transplant; HV, healthy volunteers; NfL, neurofilament light chain; OPN/SPP1, osteopontin/secreted phosphoprotein 1; sCSF1R, soluble colony-stimulating factor 1 receptor; SD, standard deviation; sTREM2, soluble triggering receptor on myeloid cells 2.

Changes in protein levels of NfL (in serum and CSF), GFAP (in serum and CSF), sCSF1R (in CSF), sTREM2 (in CSF), and OPN/SPP1 (in CSF) were evaluated. Baseline fluid biomarker levels are presented in Fig. 3 and Supplementary Table 4, and normative values in healthy volunteers (HVs) from the VGL101-01.101 study^40^ are summarized in Supplementary Table 5.

At baseline, mean ± SD serum NfL levels were significantly higher in symptomatic-no HSCT (117.4 ± 69.7 pg/ml) and symptomatic + HSCT (15.6 ± 4.73 pg/ml) subgroups compared with HVs (10.1 ± 5.5 pg/ml); the difference between HV and prodromal subgroups (27.0 ± 30.9 pg/ml) was not significant (Fig. 3A; Supplementary Table 6). Baseline NfL levels in CSF (mean ± SD, 7989.9 ± 6483.5 pg/ml) were significantly higher in the symptomatic-no HSCT subgroup compared with HVs (801 ± 1605 pg/ml) prodromal, or symptomatic HSCT subgroup (Fig. 3B; Supplementary Tables 4 and 5). Similarly, baseline levels of GFAP, a biomarker of astrogliosis, were significantly higher in both serum and CSF in the symptomatic-no HSCT subgroup compared with HV; whereas serum levels in the prodromal and symptomatic + HSCT subgroups were similar to each other and significantly elevated compared with HVs (Fig. 3C and D). Correlations between serum versus CSF levels of both NfL (*R*^2^ = 0.5785; *P* < 0.001) and GFAP (*R*^2^ = 0.2209; *P* = 0.027) at baseline suggest that serum collection only may be sufficient to assess these disease markers, relieving patients of the burden of a lumbar puncture; however, confirmation of this finding is warranted with a replicate sample. Baseline sCSF1R levels in CSF were ∼80% lower in the prodromal and symptomatic-no HSCT subgroups relative to HVs (Fig. 3E; Supplementary Tables 4 and 5). In contrast, levels in the symptomatic + HSCT subgroup were significantly higher relative to the symptomatic-no HSCT subgroup. No statistically significant differences in CSF levels of sTREM2 (Fig. 3F) and OPN/SPP1 (Fig. 3G) at baseline were observed across subgroups, but the small number of participants in the CSF substudy may limit the interpretation of these data.

**Figure 4:**
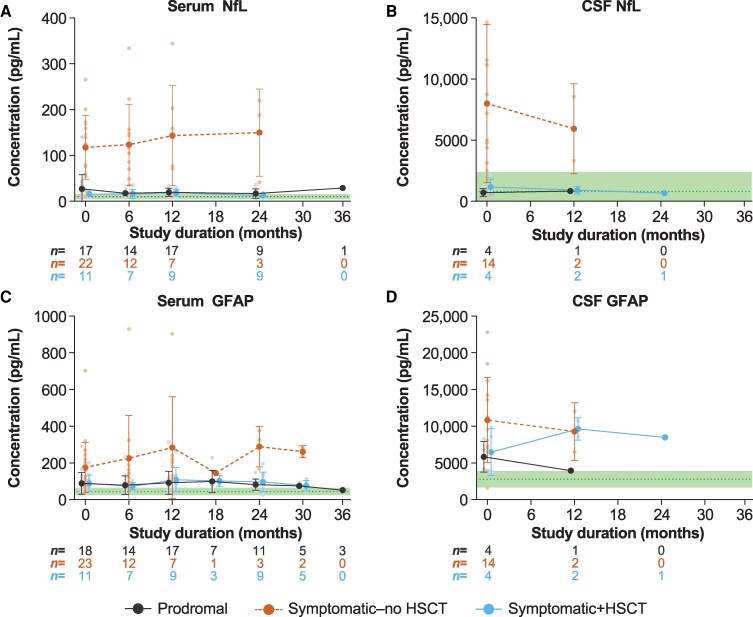
**(A) Serum NfL, (B) CSF NfL, (C) serum GFAP and (D) CSF GFAP by visit.** Solid circles and error bars represent mean and SD, respectively, and lighter-coloured circles represent individual patient values. Normative levels in healthy volunteers are from the first-in-human single-/multiple-ascending dose study of iluzanebart^40^ and are indicated as green dotted lines (mean) with light green shading (SD). CSF, cerebrospinal fluid; GFAP, glial fibrillary acidic protein; HSCT, haematopoietic stem cell transplant; NfL, neurofilament light chain; SD, standard deviation.

The elevated serum NfL levels observed at baseline (Fig. 3A) were maintained through 36 months in all subgroups compared with HVs (Fig. 4A; Supplementary Tables 4 and 5), suggesting that these elevated levels represent active neuronal injury. Elevated serum NfL levels were most apparent in the symptomatic-no HSCT subgroup, whereas levels in the prodromal and symptomatic + HSCT subgroups were comparable across all time points for which data were available. Data for NfL levels in CSF were available only for 12-month timepoint, which showed elevated levels in the symptomatic + HSCT subgroup compared with the other subgroups (Fig. 4B). Serum GFAP levels in all subgroups generally remained consistently elevated through 36 months, with increased levels most apparent in the symptomatic-no HSCT subgroup (Fig. 3C; Fig. 4C; Supplementary Tables 4 and 5), suggesting ongoing reactive astrogliosis. Similarly, sCSF1R levels in CSF remained elevated in the symptomatic + HSCT subgroup compared with the other subgroups (Fig. 4D; Supplementary Table 4).

### MRI assessments

Brain atrophy in study participants was quantified using volumetry (ventricle volume, grey matter volume, whole brain volume, and corpus callosum volume and thickness) and the Sundal MRI severity score to assess cumulative disease impact over time. At baseline, the prodromal subgroup had the least brain atrophy (Table 1; Supplementary Table 7); ventricle volume in the symptomatic-no HSCT subgroup (mean ± SD, 67.4 ± 27.0 ml) was greater relative to the prodromal subgroup (30.0 ± 15.8 ml) and greatest in the symptomatic + HSCT subgroup (117.5 ± 38.5 ml). The latter finding is likely explained by the longer disease duration in participants who received HSCT. Similar findings of increased atrophy in the symptomatic-no HSCT and symptomatic + HSCT subgroups were observed for grey matter volume, whole brain volume, and corpus callosum volume and thickness. White matter lesion volume and Sundal MRI severity scores increased remarkably from the prodromal to the symptomatic-no HSCT to the symptomatic + HSCT subgroups. Unlike the fluid biomarker analyses, normative values in HVs were not available for MRI outcomes.

**Figure 5:**
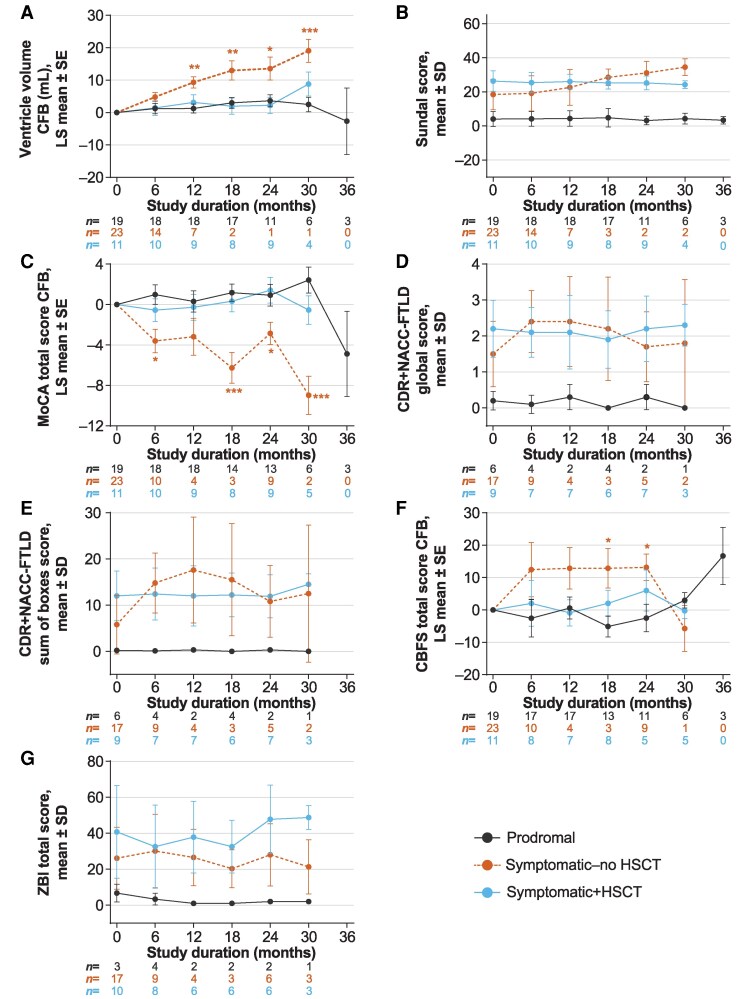
**Summary of MRI and clinical outcome assessments by visit, using a mixed model for repeated measures to estimate LS means and SEs for each subgroup^a^: (A) MRI ventricle volume,^b^ (B) Sundal MRI severity score, (C) MoCA total score,^b^ (D) CDR + NACC-FTLD global score,^c^ (E) CDR + NACC-FTLD sum of boxes score,^c^ (F) CBFS total score^b^ and (G) ZBI total score.^c^** *P < 0.05; **P < 0.01; ***P < 0.001 for difference in LS mean CFB versus the prodromal subgroup. ^a^A mixed model for repeated measures was used for estimating LS means and SEs for each subgroup; however, due to low sample size, these estimates may not be reliable and should be interpreted with caution. ^b^Early study termination and missing data limited the feasibility of a number of preplanned analyses. Therefore, statistical analysis focused on a subset of longitudinal observations only. For those outcomes for which change from baseline data were able to be calculated [MRI ventricle volume (panel A), MoCA total score (panel C), CBFS total score (panel F)], LS means and SEs for each subgroup and LS mean differences from the prodromal subgroup were calculated as described in the Methods. ^c^Missing data for the CDR + NACC-FTLD and ZBI assessments may be due to the following reasons: administration of the CDR + NACC-FTLD was at the investigator’s discretion if no signs or symptoms of cognitive impairment (i.e. normal MoCA score) were evident; neither the CDR + NACC-FTLD nor the ZBI was administered if the subject did not have a study partner who had provided informed consent for the study; and study partners were not required for prodromal participants. CBFS, Cortical Basal ganglia Functional Scale; CDR + NACC-FLTD, Clinical Dementia Rating Scale plus National Alzheimer’s Coordinating Center-Frontotemporal Lobar Degeneration; CFB, change from baseline; HSCT, haematopoietic stem cell transplant; LS, least squares; MoCA, Montreal Cognitive Assessment; MRI , magnetic resonance imaging; SD, standard deviation; SE, standard error; ZBI, Zarit Burden Interview.

Compared with MRI outcomes at baseline, the greatest change in ventricle volume was observed in the symptomatic-no HSCT subgroup (Fig. 5A). Sundal MRI severity scores were highest among both symptomatic subgroups throughout the study compared with the prodromal subgroup (Fig. 5B).

### Clinical assessments

The MoCA and CDR + NACC-FTLD instruments were used to evaluate participants’ cognitive abilities and dementia severity. At baseline, participants in the prodromal subgroup had normal cognitive abilities (mean ± SD MoCA total score, 27.4 ± 1.9), whereas the symptomatic subgroups had mild cognitive impairment at baseline (symptomatic-no HSCT, 19.7 ± 6.1; symptomatic + HSCT, 18.7 ± 5.9) (Table 1), consistent with the greater brain atrophy observed for these subgroups. Over time, the MoCA total score declined in the symptomatic-no HSCT subgroup, suggesting worsening cognition throughout the study (Fig. 5C), whereas minimal changes in MoCA total scores were observed in the prodromal and symptomatic + HSCT subgroups. Similarly, CDR + NACC-FTLD global (Fig. 5D) and sum of boxes (Fig. 5E) scores were lowest in the prodromal subgroup, compared with both symptomatic subgroups, and remained stable throughout the study.

The CBFS was used to evaluate participants’ experiences with daily living and behavioural, language, and cognitive impairments. Minimal changes from baseline in CBFS total scores were observed for the prodromal and symptomatic + HSCT subgroups (Fig. 5F). In the symptomatic-no HSCT subgroup, CBFS total scores appeared to worsen from baseline to month 24.

The ZBI was used to assess the burden of CSF1R-ALSP on patients’ primary caregivers. ZBI total scores were lowest in the prodromal subgroup compared with the symptomatic subgroups (Fig. 5G). ZBI total scores were highest in the symptomatic + HSCT subgroup at baseline but were comparable to those in the symptomatic-no HSCT subgroup from month 6 through month 18.

**Figure 6:**
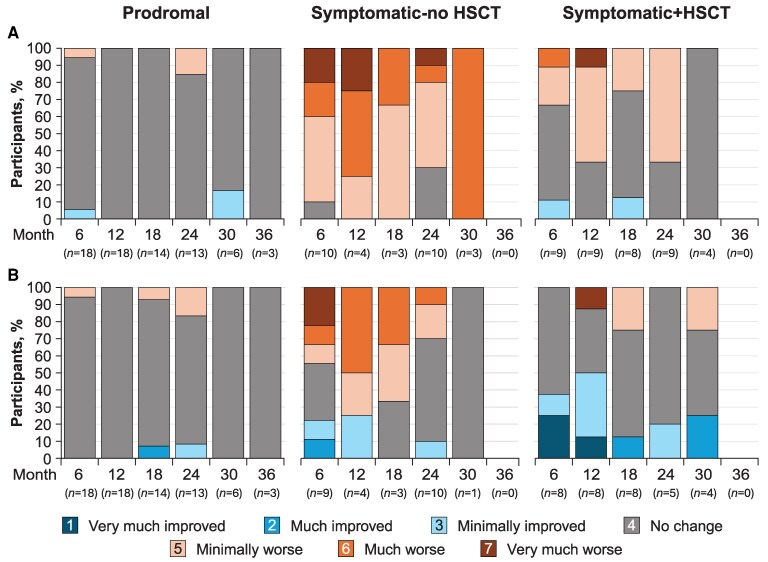
**Summary of (A) CGI-C and (B) PGI-C assessments by visit for the prodromal subgroup (left panel), symptomatic-no HSCT subgroup (middle panel), and symptomatic + HSCT subgroup (right panel).** The Clinical Global Impression–Severity of Change and Patient Global Impression–Severity of Change scales were used to assess change from baseline in participants’ overall condition. For CGI-C and PGI-C, the clinician and patient, respectively, rated the participant’s overall condition on a 7-point scale that varied from 1 = very much improved to 7 = very much worse. CGI-C and PGI-C were completed at months 6, 12, 18, 24, 30 and 36/ET. The staked bar graphs represent the percentage of patients with each score at each time point. CGI-C, Clinical Global Impression–Severity of Change; ET, end of treatment; HSCT, haematopoietic stem cell transplant; PGI-C, Patient Global Impression–Severity of Change.

Clinicians used the CGI-S to qualitatively assess baseline disease severity and the CGI-C to assess change from baseline in participants’ overall condition relative to past experiences in treating patients with CSF1R-ALSP; the PGI-S and PGI-C, respectively, evaluated the same outcomes from the patients’ perspective. Supplementary Fig. 2 summarizes baseline CGI-S and PGI-S scores. Over the course of the study, few changes in CGI-C or PGI-C were observed in the prodromal subgroup. In contrast, CGI-C and PGI-C scores in the symptomatic subgroups worsened over time, most prominently among those in the symptomatic-no HSCT subgroup (Fig. 6).

### Correlations between brain atrophy and clinical outcomes

Because MRI measures of brain volume appeared to track with disease severity at baseline and demonstrated the greatest longitudinal change over time relative to other biomarkers, the relationship between brain volume loss and clinical measures was examined using Pearson correlation analyses at baseline and longitudinally over time (i.e. change from baseline to 12 months). At baseline, moderate associations were observed between ventricle volume and MoCA total score (Supplementary Fig. 3), CBFS total score, CDR-NACC + FTLD-SB and CGI-S, demonstrating that greater ventricle volume was associated with greater cognitive impairment (Table 2). A consistent pattern was observed for all measures of brain volume loss assessed, with greater white matter lesion volume, reduced grey matter volume, and reduced corpus callosum volume associated with greater cognitive impairment (Supplementary Figs 4 and 5). Longitudinally, greater brain volume loss was associated with greater clinical worsening, with the strongest correlations observed for ventricle volume and grey matter volume measurements (Table 2). A number of these correlations remained significant after correcting for multiple comparisons.

**Table 2:** Pearson correlation analyses between MRI brain atrophy or fluid biomarkers and clinical assessments

MRI assessment or fluid biomarker	Clinical outcome assessment											
	MoCA total score			CBFS total score			CDR + NACC-FTLD-SB score			CGI-S/CGI-C^a^		
	*n*	*r*	*P* value^b^	*n*	*r*	*P* value^b^	*n*	*r*	*P* value^b^	*n*	*r*	*P* value^b^
**Baseline correlations^c^**												
Ventricle volume	53	−0.54	<0.001^d^	**53**	**0**.**41**	**0**.**002**	**32**	**0**.**52**	**0**.**002**	**53**	**0**.**68**	**<0**.**001**^d^
Total grey matter volume	**53**	**0**.**45**	**<0**.**001**^d^	53	−0.38	0.005	32	−0.27	0.137	53	−0.53	<0.001^d^
Corpus callosum volume	**53**	**0**.**57**	**<0**.**001**^d^	53	−0.56	<0.001^d^	32	−0.56	0.001	53	−0.65	<0.001^d^
Corpus callosum thickness	**53**	**0**.**62**	**<0**.**001**^d^	53	−0.54	<0.001^d^	32	−0.56	<0.001^d^	53	−0.75	<0.001^d^
White matter lesion volume	53	−0.48	<0.001^d^	**53**	**0**.**53**	**<0**.**001**^d^	**32**	**0**.**40**	**0**.**023**	**53**	**0**.**65**	**<0**.**001**^d^
NfL (serum)	50	−0.43	0.002	**50**	**0**.**51**	**<0**.**001**^d^	**31**	**0**.**30**	**0**.**101**	**50**	**0**.**34**	**0**.**015**
GFAP (serum)	52	−0.34	0.015	**52**	**0**.**38**	**0**.**006**	**32**	**0**.**26**	**0**.**147**	**52**	**0**.**32**	**0**.**020**
**Longitudinal (month 12 CFB) correlations^c^**												
Ventricle volume	31	−0.64	<0.001^d^	**28**	**0**.**55**	**0**.**002**	**12**	**0**.**73**	**0**.**007**	**31**	**0**.**76**	**<0**.**001**^d^
Total grey matter volume	**31**	**0**.**70**	**<0**.**001**^d^	28	−0.45	0.017	12	−0.58	0.049	31	−0.76	<0.001^d^
Corpus callosum volume	**31**	**0**.**16**	**0**.**379**	28	0.10	0.609	12	0.16	0.616	31	−0.23	0.221
Corpus callosum thickness	**31**	**0**.**19**	**0**.**298**	28	−0.12	0.555	12	−0.26	0.416	31	−0.35	0.053
White matter lesion volume	31	−0.29	0.117	**28**	**0**.**52**	**0**.**004**	**12**	**0**.**63**	**0**.**029**	**31**	**0**.**24**	**0**.**189**
NfL (serum)	29	−0.51	0.005	**26**	**0**.**49**	**0**.**011**	**12**	**0**.**61**	**0**.**034**	**29**	**0**.**49**	**0**.**007**
GFAP (serum)	29	−0.59	<0.001^d^	**26**	**0**.**66**	**<0**.**001**^d^	**12**	**0**.**46**	**0**.**137**	**29**	**0**.**43**	**0**.**019**

^a^Outcome was CGI-S for baseline correlations and CGI-C for month 12 CFB correlations.

^b^ *P* values are unadjusted.

^c^Correlations expected to be directly proportional (both parameters change in the same direction) are indicted with bold text; correlations expected to be inversely proportional (parameters change in opposite directions) are indicated with nonbold text.

^d^Significant at an adjusted alpha of *P* < 0.0009 based on Bonferroni correction.

CBFS, Cortical Basal ganglia Functional Scale; CDR + NACC-FTLD-SB, Clinical Dementia Rating Scale plus National Alzheimer’s Coordinating Center-Frontotemporal Lobar Degeneration sum of boxes; CFB, change from baseline; CGI-C, Clinical Global Impression of Change; CGI-S, Clinical Global Impression–Severity of Illness; GFAP, glial fibrillary acidic protein; MoCA, Montreal Cognitive Assessment; MRI, magnetic resonance imaging; *n*, number of pairs; NfL, neurofilament light chain; *r*, sample correlation coefficient.

### Correlations between serum NfL, serum GFAP and clinical outcomes

Pearson correlations between serum biomarkers of neurodegeneration and astrogliosis and clinical outcomes were also investigated (Table 2; Supplementary Figs 6 and 7). At baseline, significant (*P* < 0.05) but weak correlations were observed between serum NfL and the clinical outcomes of MoCA total score, CBFS total score and CGI-S, but only the correlation between serum NfL and CBFS total score was significant after correction for multiple comparisons. A similar pattern was observed with serum GFAP, but no correlations survived correction. Longitudinally, only the correlations between serum GFAP and MoCA total score and CBFS total score survived correction for multiple comparisons. Overall, the correlations of clinical outcomes with fluid biomarkers were weaker than those observed with measures of brain atrophy.

## Discussion

In this first of its kind, prospective natural history study of CSF1R-ALSP, biomarker, radiologic and clinical characteristics of the disease were systematically assessed in a cohort of 53 individuals with autosomal-dominant *CSF1R* variants. The study population was broadly representative of the CSF1R-ALSP population described in the literature, with participants on average in their mid-40s at the time of diagnosis and the clinical assessments demonstrating both cognitive and motor impairments, reflecting the characteristic multisystem neurological involvement of CSF1R-ALSP.^4,7,41^

Substantial genetic variability in the pathogenic *CSF1R* gene, encompassing 37 distinct variants, was observed in this cohort; this precluded meaningful comparison of the effects of individual mutations on the clinical and radiological course of CSF1R-ALSP. Consequently, the analysis focused on larger cohorts of prodromal and symptomatic patients, rather than on individual disease trajectories, which could be confounded by genetic diversity. Findings from these analyses suggest that prodromal *CSF1R* variant carriers may remain relatively stable over time (up to 36 months), with little change in serum, CSF or imaging biomarkers and stable neurological function and cognitive assessments. Undergoing HSCT appears to slow disease progression; participants in this study who had received HSCT before enrolment did not show a significant decline over the course of the trial, as measured by imaging and clinical markers as well as by serum and CSF NfL levels.

This study examined several biomarkers pertinent to the CSF1R-ALSP disease process that may aid in future drug development. CSF and serum NfL levels appeared to be elevated in symptomatic participants relative to prodromal and HVs, consistent with observations in other rapidly progressive neurodegenerative diseases such as amyotrophic lateral sclerosis and indicating active neuronal degeneration.^42-46^ Regarding other fluid biomarkers, *CSF1R* variant carriers in the prodromal stages had few clinical symptoms but had reduced CSF sCSF1R levels at baseline compared with normative levels in HVs. This might suggest that the presence of a *CSF1R* variant is associated with measurably lower mean soluble protein product of that gene (Fig. 3) and that an additional environmental or genetic factor may be needed to trigger symptomatic disease progression in *CSF1R* variant carriers. CSF levels of TREM2, another microglial receptor, appeared to be generally comparable across subgroups, disease stage and HVs. The average levels of serum and CSF GFAP, a marker of astrogliosis,^47^ were elevated in *CSF1R* variant carriers, particularly those in the symptomatic-no HSCT subgroup.

Differences in imaging biomarkers were observed at baseline among participants in the prodromal, symptomatic-no HSCT and symptomatic + HSCT subgroups. Ventricle volume and white matter lesion volume, MRI measures that increase with increasing brain atrophy, were lowest in prodromal participants and higher in symptomatic participants (with or without a history of HSCT), tracking with disease stage.^15^ MRI measures of brain tissue volume (e.g. grey matter, corpus callosum and whole brain volumes), which decrease with increasing brain atrophy, were lowest in symptomatic participants (with or without a history of HSCT) and greatest in prodromal participants. Within the symptomatic subgroups, the radiological differences between those with versus without a history of HSCT suggest that participants who underwent HSCT had likely already experienced overt disease manifestation and more advanced disease progression, including higher white matter lesion burden and greater cerebral atrophy. The semiquantitative, radiologist-determined Sundal MRI severity score also demonstrated a consistent pattern, with symptomatic participants (with or without a history of HSCT) demonstrating scores that were 4‒6 times higher than in the prodromal subgroup. The latter data support that the Sundal MRI severity score may be a practical tool for disease staging in clinical practice.^12^ Thus, despite the increased sCSF1R levels in CSF of the symptomatic + HSCT subgroup versus the other subgroups, imaging parameters in the symptomatic + HSCT subgroup were the most severe at baseline but stable over the course of the study. Longitudinally, MRI ventricle volume increased over the duration of the study to a significantly greater extent in the symptomatic-no HSCT subgroup compared with the overlapping profiles observed for the other two subgroups, which remained relatively stable over the course of the 24 months for which sufficient observations were available (Fig. 5A). Of note, detailed information about patients treated with HSCT, regarding the timing of the intervention, protocol and clinical rationale was not available as the therapy was performed outside the study’s observation period, limiting the interpretability of outcome comparisons.

Cognitive function as measured by the MoCA score was best in the prodromal subgroup; similar patterns were observed for the CDR + NACC-FTLD and CBFS outcomes: scores in the prodromal subgroup were near normal, whereas functional impairment was evident from the symptomatic-no HSCT and symptomatic + HSCT subgroup scores. Motor function, as assessed by the 2MWT and the TUG, and disease burden, as assessed by the FAQ and ZBI, were also most mildly impacted in prodromal participants. In the longitudinal clinical outcome findings shown in Fig. 5, variable point estimates of the mean with high SDs are apparent. Of the clinical outcome measures for which longitudinal data were available, only the MoCA in symptomatic-no HSCT participants showed a clear pattern of decline.

The clinician and patient/caregiver severity of illness assessments showed milder disease severity in the prodromal subgroup both at baseline (Supplementary Fig. 1) and over time (Fig. 6). The symptomatic-no HSCT subgroup comprised a mix of mild, moderate and severe baseline assessments that progressed towards more severe disease stages over time; for the symptomatic + HSCT subgroup, a range of disease stages from mild to severe was seen at baseline and remained mixed over the course of the study. The patient/caregiver severity ratings mirrored the clinician assessments (Fig. 6; Supplementary Fig. 1), except for the symptomatic + HSCT subgroup, in which patients/caregivers rated disease as mild more frequently than clinicians. This discrepancy may be addressed with further research into the patient experience of living with CSF1R-ALSP and application of outcome measures that are relevant to the patient experience.

An important result from this study is the associations observed between MRI measures of brain volume loss and clinical outcome measures (Table 2). These correlations were present across multiple measures of brain volume loss, including ventricle volume, grey matter volume, corpus callosum volume and thickness, and white matter lesion volume, and demonstrated associations with cognitive function, as assessed by the MoCA, as well as with CBFS, CDR + NACC-FTLD and CGI-C. Although multiplicity correction resulted in few correlations meeting the adjusted significance level, correlations with *P* < 0.0009, such as that observed between ventricle volume and MoCA total score, are sufficiently robust to support a relationship between MRI brain volume loss and cognitive decline. Consistency of the relationship across multiple measures of brain volume loss pertinent to CSF1R-ALSP further supports this relationship. Correlations were observed not only cross-sectionally at baseline but also over time, as evidenced by the longitudinal correlations between rates of change for imaging and clinical measures. MRI brain volume loss is a potential biomarker that may be useful in future drug development for CSF1R-ALSP.

The symptomatic, untreated patients with CSF1R-ALSP show progressive clinical decline and brain atrophy on MRI that are very similar to patterns observed in other neurodegenerative diseases,^28,29^ although the magnitude and rate of radiological progression are greater in CSF1R-ALSP (Supplementary Table 8). When compared against progressive multiple sclerosis and Alzheimer’s disease, patients with symptomatic CSF1R-ALSP showed 1.2- and 1.4-fold greater baseline atrophy, respectively, and 2.5- and 2.0-fold higher rates of progression over time. The comparison is even more striking against the less impaired populations of mild cognitive impairment and normal elderly controls, with rates of ventricle volume expansion in CSF1R-ALSP that were 3.4- and 8.5-fold faster, respectively. These differentials suggest an additional driver of brain volume loss (e.g. grey matter loss) besides the neurodegeneration in CSF1R-ALSP due to white matter pathology. Literature on other leukodystrophies is limited; however, although the ventricle volume enlargement due to tissue loss is not specific to a particular neurodegenerative disease, the degree of neurodegeneration at presentation and its progression as measured by MRI brain volume loss is remarkably greater in the CSF1R-ALSP disease process than that in Alzheimer’s disease and multiple sclerosis, and certainly occurs at a younger age in CSF1R-ALSP than in Alzheimer’s disease.

This study provided several learning opportunities. The fluid biomarker results indicate ongoing neuronal (persistently elevated NfL) and astrocyte (persistently elevated GFAP) degeneration in symptomatic participants. In addition to characterizing the natural history of CSF1R-ALSP, this study identified relatively noninvasive biomarkers of both disease severity (e.g. volumetric MRI measures, Sundal MRI severity score, serum NfL and GFAP levels) and disease progression (e.g. volumetric MRI measures), as well as clinical measures that are sensitive to disease progression (e.g. MoCA, CDR + NACC-FTLD), and will be of value for future therapeutic intervention trials.

All clinical outcomes assessed here appeared feasible in this population. However, the study teams observed some distress and burden on the part of patients and caregivers when many assessments were performed in a single study visit or were administered frequently enough that recall from previous visits may have influenced patient/caregiver responses. Some clinical outcome measures might have benefited from a pre-specified requirement for caregiver participation. These limitations would ideally be addressed in collaboration with the patient community in future studies to optimize study protocol and minimize burden. Building upon the experience of this study, the CSF1R-ALSP research community may also be able to develop more tailored clinical assessments that better reflect the broad phenotype and progressive nature of this disease.

Our study has several other limitations. Subgroup comparisons were limited by the relatively small group sizes, particularly CSF biomarker analysis, since participation in the CSF substudy was optional. Small sample size precluded statistical comparisons; therefore, generalizability of these data should be confirmed in future studies with larger sample sizes. The correlation analyses were similarly limited and did not allow for controlling all variables of interest, such as age and sex. Finally, patients with CSF1R-ALSP who participated in this study may not be representative of the underlying CSF1R-ALSP population in their access to healthcare, education or socioeconomic status.

Our results are consistent with previously reported case reports and case series that have suggested that HSCT can have potential benefit in CSF1R-ALSP.^18-25^ We found that imaging (MRI), fluid biomarker (NfL) and clinical assessments were stable in participants who had undergone HSCT. However, our conclusions on this subgroup are limited because participants were not observed before the HSCT procedure but rather >6 months after transplant. The post-HSCT group described here is a selected cohort and subject to bias, as it does not reflect outcomes in others with CSF1R-ALSP who have received HSCT but were not well enough to later participate in a research study. Because of this inherent limitation, future longitudinal studies of patients with CSF1R-ALSP, both before and after HSCT, are important to assess the full impact of HSCT in this disease.

This prospective natural history study represents the largest and most comprehensive evaluation of patients with CSF1R-ALSP to date. The findings confirm that disease progression is slow in the prodromal phase but relentless once symptoms manifest, with marked and measurable decline observed across cognitive, motor and imaging domains. Importantly, this study demonstrates for the first time which fluid and imaging biomarkers robustly track clinical disease status and progression, highlighting their potential roles not only in diagnosis and prognostication but also as candidate endpoints in future clinical trials. The next critical step will be to determine whether therapeutic interventions that modify these biomarkers can also change clinical outcomes and, conversely, whether clinical improvement is reflected in biomarker trajectories.

## Supplementary Material

fcag271_Supplementary_Data

## Data Availability

Anonymized patient data and related study materials that support the findings of this article cannot be made publicly available at the time of publication due to evolving commercial agreements. However, investigator teams at each study site have access to data for the study participants from their site, and data will be made available to qualified investigators upon reasonable request to primary investigators at each site (Supplementary Table 1).

## References

[1] Papapetropoulos S, Pontius A, Finger E, et al Adult-onset leukoencephalopathy with axonal spheroids and pigmented glia: Review of clinical manifestations as foundations for therapeutic development. Front Neurol. 2022;12:788168. 35185751 10.3389/fneur.2021.788168 PMC8850408

[2] Rademakers R, Baker M, Nicholson AM, et al Mutations in the colony stimulating factor 1 receptor (*CSF1R*) gene cause hereditary diffuse leukoencephalopathy with spheroids. Nat Genet. 2011;44(2):200–205. 22197934 10.1038/ng.1027 PMC3267847

[3] Schmitz AS, Raju J, Köhler W, et al Novel variants in CSF1R associated with adult-onset leukoencephalopathy with axonal spheroids and pigmented glia (ALSP). J Neurol. 2024;271(9):6025–6037. 39031193 10.1007/s00415-024-12557-0 PMC11377666

[4] Wade C, Runeckles K, Chataway J, Houlden H, Lynch DS. *CSF1R*-related disorder: Prevalence of *CSF1R* variants and their clinical significance in the UK population. Neurol Genet. 2024;10(4):e200179. 39040919 10.1212/NXG.0000000000200179 PMC11261581

[5] Konno T, Kasanuki K, Ikeuchi T, Dickson DW, Wszolek ZK. *CSF1R*-related leukoencephalopathy: A major player in primary microgliopathies. Neurology. 2018;91(24):1092–1104. 30429277 10.1212/WNL.0000000000006642 PMC6329328

[6] Dulski J, Sundal C, Wszolek ZK. *CSF1R*-related Disorder. In: Adam MP, Feldman J, Mirzaa GM, Pagon RA, Wallace SE, Amemiya A, eds. Genereviews®. University of Washington; 2012. 22934315

[7] Konno T, Yoshida K, Mizuno T, et al Clinical and genetic characterization of adult-onset leukoencephalopathy with axonal spheroids and pigmented glia associated with *CSF1R* mutation. Eur J Neurol. 2017;24(1):37–45. 27680516 10.1111/ene.13125 PMC5215554

[8] Papapetropoulos S, Gelfand JM, Konno T, et al Clinical presentation and diagnosis of adult-onset leukoencephalopathy with axonal spheroids and pigmented glia (ALSP): A literature analysis of case studies. Front Neurol. 2024;15:1320663. 38529036 10.3389/fneur.2024.1320663 PMC10962389

[9] Rutherford HA, Rush BK, Smith A, et al Mapping the journey of patients and care partners living with adult-onset leukoencephalopathy with axonal spheroids and pigmented glia: Developing a framework for improvements in care. Neurodegener Dis Manag. 2024;14(5):161–172. 39363647 10.1080/17582024.2024.2404378 PMC11524202

[10] ICD-10 Data. 2025 ICD-10-CM diagnosis code G93.44: Adult-onset leukodystrophy with axonal spheroids. Accessed 10 July 2025. https://www.icd10data.com/ICD10CM/Codes/G00-G99/G89-G99/G93-/G93.44

[11] Sister’s Hope Foundation. Understanding ALSP: Genetic testing. Accessed 10 July 2025. https://www.sistershopefoundation.org/genetic-testing

[12] Sundal C, Van Gerpen JA, Nicholson AM, et al MRI characteristics and scoring in HDLS due to CSF1R gene mutations. Neurology. 2012;79(6):566–574. 22843259 10.1212/WNL.0b013e318263575a PMC3413763

[13] Van Gerpen JA, Wider C, Broderick DF, Dickson DW, Brown LA, Wszolek ZK. Insights into the dynamics of hereditary diffuse leukoencephalopathy with axonal spheroids. Neurology. 2008;71(12):925–929. 18794495 10.1212/01.wnl.0000325916.30701.21 PMC2843529

[14] Rajagovindan R, O’Mara R, Meier A, et al Radiological features of adult-onset leukoencephalopathy with axonal spheroids and pigmented glia (ALSP) and its longitudinal progression (P8-4.003). Neurology. 2023;100(17_supplement_2):3230.

[15] Kinoshita M, Oyanagi K, Matsushima A, et al Adult-onset leukoencephalopathy with axonal spheroids and pigmented glia (ALSP): Estimation of pathological lesion stage from brain images. J Neurol Sci. 2024;461:123027. 38805875 10.1016/j.jns.2024.123027

[16] Sundal C, Lash J, Aasly J, et al Hereditary diffuse leukoencephalopathy with axonal spheroids (HDLS): A misdiagnosed disease entity. J Neurol Sci. 2012;314(1-2):130–137. 22050953 10.1016/j.jns.2011.10.006 PMC3275663

[17] National Organization for Rare Disorders (NORD) . Adult-onset leukoencephalopathy with axonal spheroids and pigmented glia. Updated 19 February 2025. Accessed 10 June 2025. https://rarediseases.org/rare-diseases/adult-onset-leukoencephalopathy-with-axonal-spheroids-and-pigmented-glia/

[18] Eichler FS, Li J, Guo Y, et al CSF1R mosaicism in a family with hereditary diffuse leukoencephalopathy with spheroids. Brain. 2016;139(Pt 6):1666–1672. 27190017 10.1093/brain/aww066 PMC4892751

[19] Gelfand JM, Greenfield AL, Barkovich M, et al Allogeneic HSCT for adult-onset leukoencephalopathy with spheroids and pigmented glia. Brain. 2020;143(2):503–511. 31840744 10.1093/brain/awz390

[20] Tipton PW, Kenney-Jung D, Rush BK, et al Treatment of CSF1R-related leukoencephalopathy: Breaking new ground. Mov Disord. 2021;36(12):2901–2909. 34329526 10.1002/mds.28734

[21] Dulski J, Heckman MG, White LJ, Żur-Wyrozumska K, Lund TC, Wszolek ZK. Hematopoietic stem cell transplantation in CSF1R-related leukoencephalopathy: Retrospective study on predictors of outcomes. Pharmaceutics. 2022;14(12):2778. 36559271 10.3390/pharmaceutics14122778 PMC9788080

[22] Yska HAF, Golse M, Beerepoot S, et al Hematopoietic stem cell transplantation in an international cohort of colony stimulating factor-1 receptor (CSF1R)-related disorder. Mov Disord. 2025;40(9):1826–1835. 40646711 10.1002/mds.30282 PMC12485588

[23] Mochel F, Delorme C, Czernecki V, et al Haematopoietic stem cell transplantation in *CSF1R*-related adult-onset leukoencephalopathy with axonal spheroids and pigmented glia. J Neurol Neurosurg Psychiatry. 2019;90(12):1375–1376. 31213485 10.1136/jnnp-2019-320701

[24] Han J, Sarlus H, Wszolek ZK, Karrenbauer VD, Harris RA. Microglial replacement therapy: A potential therapeutic strategy for incurable CSF1R-related leukoencephalopathy. Acta Neuropathol Commun. 2020;8(1):217. 33287883 10.1186/s40478-020-01093-3 PMC7720517

[25] Wu J, Wang Y, Li X, et al Microglia replacement halts the progression of microgliopathy in mice and humans. Science. 2025;389(6756):eadr1015. 10.1126/science.adr1015 40638739

[26] Khaddour K, Hana CK, Mewawalla P. Hematopoietic stem cell transplantation. Statpearls. StatPearls Publishing; 2023. 30725636

[27] Vigil Neuroscience Inc. Vigil Neuroscience provides update on iluzanebart phase 2 IGNITE trial in ALSP. Accessed 1 July 2025. https://www.globenewswire.com/news-release/2025/06/04/3093434/0/en/vigil-neuroscience-provides-update-on-iluzanebart-phase-2-ignite-trial-in-alsp.html

[28] Genovese AV, Hagemeier J, Bergsland N, et al Atrophied brain T2 lesion volume at MRI is associated with disability progression and conversion to secondary progressive multiple sclerosis. Radiology. 2019;293(2):424–433. 31549947 10.1148/radiol.2019190306 PMC6823621

[29] Nestor SM, Rupsingh R, Borrie M, et al Ventricular enlargement as a possible measure of Alzheimer’s disease progression validated using the Alzheimer’s disease neuroimaging initiative database. Brain. 2008;131(Pt 9):2443–2454. 18669512 10.1093/brain/awn146 PMC2724905

[30] A natural history study of patients with adult-onset leukoencephalopathy with axonal spheroids and pigmented glia (ALSP). Recruiting, Vigil Neuroscience, Inc. Accessed 1 July 2025. https://clinicaltrials.gov/study/NCT05020743

[31] Nasreddine ZS, Phillips NA, Bédirian V, et al The Montreal Cognitive Assessment, MoCA: A brief screening tool for mild cognitive impairment. J Am Geriatr Soc. 2005;53(4):695–699. 15817019 10.1111/j.1532-5415.2005.53221.x

[32] Smith T, Gildeh N, Holmes C. The Montreal cognitive assessment: Validity and utility in a memory clinic setting. Can J Psychiatry. 2007;52(5):329–332. 17542384 10.1177/070674370705200508

[33] Miyagawa T, Brushaber D, Syrjanen J, et al Use of the CDR® plus NACC FTLD in mild FTLD: Data from the ARTFL/LEFFTDS consortium. Alzheimers Dement. 2020;16(1):79–90. 31477517 10.1016/j.jalz.2019.05.013 PMC6949373

[34] Witherspoon JW, Vasavada R, Logaraj RH, et al Two-minute versus 6-minute walk distances during 6-minute walk test in neuromuscular disease: Is the 2-minute walk test an effective alternative to a 6-minute walk test? Eur J Paediatr Neurol. 2019;23(1):165–170. 30449663 10.1016/j.ejpn.2018.10.001 PMC6423958

[35] Ibrahim A, Singh DKA, Shahar S, Omar MA. Timed up and go test combined with self-rated multifactorial questionnaire on falls risk and sociodemographic factors predicts falls among community-dwelling older adults better than the timed up and go test on its own. J Multidiscip Healthc. 2017;10:409–416. 29138571 10.2147/JMDH.S142520 PMC5667639

[36] Pfeffer RI, Kurosaki TT, Harrah CH Jr., Chance JM, Filos S. Measurement of functional activities in older adults in the community. J Gerontol. 1982;37(3):323–329. 7069156 10.1093/geronj/37.3.323

[37] Knight BG, Fox LS, Chou CP. Factor structure of the burden interview. J Clin Geropyschol. 2000;6(4):249–258.

[38] Guy W . National institute of mental health, psychopharmacology research branch, early clinical drug evaluation program, In: ECDEU Assessment manual for psychopharmacology. Rev. ed. DHEW publication; no (ADM) :76-338. U. S. Dept. of Health, Education, and Welfare, Public Health Service, Alcohol, Drug Abuse, and Mental Health Administration, National Institute of Mental Health, Psychopharmacology Research Branch, Division of Extramural Research Programs; 1976.

[39] Lang AE, Stebbins GT, Wang P, et al The cortical basal ganglia functional scale (CBFS): Development and preliminary validation. Parkinsonism Relat Disord. 2020;79:121–126. 32947108 10.1016/j.parkreldis.2020.08.021

[40] Meier A, Papapetropoulos S, Marsh A, et al Phase 1, first-in-human, single-/multiple-ascending dose study of iluzanebart in healthy volunteers. Ann Clin Transl Neurol. 2025;12(5):1065–1076. 40166927 10.1002/acn3.70033 PMC12093347

[41] Dulski J, Baker M, Banks SA, et al Global presence and penetrance of *CSF1R*-related disorder. Neurol Genet. 2024;10(5):e200187. 39280886 10.1212/NXG.0000000000200187 PMC11398975

[42] Behzadi A, Pujol-Calderón F, Tjust AE, et al Neurofilaments can differentiate ALS subgroups and ALS from common diagnostic mimics. Sci Rep. 2021;11(1):22128. 34764380 10.1038/s41598-021-01499-6 PMC8585882

[43] Zetterberg H, Skillback T, Mattsson N, et al Association of cerebrospinal fluid neurofilament light concentration with Alzheimer disease progression. JAMA Neurol. 2016;73(1):60–67. 26524180 10.1001/jamaneurol.2015.3037 PMC5624219

[44] Stilund M, Gjelstrup MC, Petersen T, Moller HJ, Rasmussen PV, Christensen T. Biomarkers of inflammation and axonal degeneration/damage in patients with newly diagnosed multiple sclerosis: Contributions of the soluble CD163 CSF/serum ratio to a biomarker panel. PLoS One. 2015;10(4):e0119681. 25860354 10.1371/journal.pone.0119681 PMC4393241

[45] Byrne LM, Rodrigues FB, Johnson EB, et al Evaluation of mutant huntingtin and neurofilament proteins as potential markers in Huntington’s disease. Sci Transl Med. 2018;10(458):eaat7108. 10.1126/scitranslmed.aat7108 30209243

[46] Meeter LH, Dopper EG, Jiskoot LC, et al Neurofilament light chain: A biomarker for genetic frontotemporal dementia. Ann Clin Transl Neurol. 2016;3(8):623–636. 27606344 10.1002/acn3.325 PMC4999594

[47] Hol EM, Pekny M. Glial fibrillary acidic protein (GFAP) and the astrocyte intermediate filament system in diseases of the central nervous system. Curr Opin Cell Biol. 2015;32:121–130. doi:10.1016/j.ceb.2015.02.004 25726916

