# Supplementary material for: The ILLUMINATE natural history study in colony-stimulating factor 1 receptor-related adult-onset leukoencephalopathy with axonal spheroids and pigmented glia
Source: Brain Commun. 2026 Jul 14;8(4):fcag271. doi: 10.1093/braincomms/fcag271 (PMC13397125; doi:10.1093/braincomms/fcag271)
Supplement: fcag271_Supplementary_Data [file fcag271_supplementary_data.docx]

**Supplementary Materials**

The Illuminate Natural History Study in colony-stimulating factor 1 receptor‒related adult-onset leukoencephalopathy with axonal spheroids and pigmented glia

**Supplementary Methods**

*Quantification of fluid biomarkers*

Prior to sample analysis, performance of each commercially available assay was validated for robustness, precision, limits of quantification, dilutional linearity, parallelism, recovery, stability, and selectivity. Acceptance criteria for each assay were defined and monitored to ensure consistency of results (**Supplementary Table 2**).

Neurofilament light chain (NfL) concentrations in serum and cerebrospinal fluid (CSF) were quantified using the human NfL Simple Plex assay (ProteinSimple, San Jose, CA, USA) employed on the Ella^TM^ automated immunoassay platform (ProteinSimple) according to the manufacturer’s instructions, with calibration performed using the in-cartridge factory standard curve. A dilution of 1:2 was used for serum and CSF samples. For each serum sample, duplicate wells were used to yield 6 replicate assay results; for each CSF sample, the architecture of the microfluidic platform enabled use of a single well to yield triplicate assay results. The quantitative range of the assay was 2.7‒10,290 pg/mL. Using endogenous quality control serum samples, the lowest serum endogenous level of quantitation was established from parallelism data as 6.82 pg/mL, and the highest endogenous upper limit of quantification was established as 136 pg/mL. Using endogenous quality control CSF samples, CSF endogenous lower limit of quantitation was established as 148 pg/mL, and endogenous upper limit of quantification was established as 14,976 pg/mL. Concentrations were interpolated from the standard using a five-parameter logistic curve fit, with 1/y^2^ weighting and adjusted for dilution factor.

Glial fibrillary acidic protein (GFAP) concentrations in serum and CSF were quantified using the ultrasensitive electrochemiluminescence immunoassay Meso Scale Discovery S-PLEX^®^ platform (Meso Scale Diagnostics, Rockville, MD, USA), as per manufacturer’s instructions. Dilutions of 1:8 and 1:32 were used for serum and CSF samples, respectively, in Diluent 63 (Meso Scale Diagnostics). For each serum and CSF sample, GFAP concentrations were measured in duplicate wells. The quantitative range of the assay was determined to be 0.51‒781 pg/mL. Using endogenous quality control serum samples, the serum endogenous lower limit of quantitation was established as 21.5 pg/mL, and the endogenous upper limit of quantification was established as 110 pg/mL. Using endogenous quality control CSF samples, CSF endogenous lower limit of quantitation was established as 1049 pg/mL, and endogenous upper limit of quantification was established as 4305 pg/mL.

CSF levels of soluble triggering receptor on myeloid cells 2 (sTREM2) were determined using DuoSet^®^ ELISA (R&D Systems, Minneapolis, MN, USA) according to manufacturer’s instructions. The CSF samples were diluted at 1:5 and tested in duplicate. The quantitative range of the assay was determined to be 9.96‒6080 pg/mL. Using endogenous quality control CSF samples, CSF endogenous lower limit of quantitation was established as 230 pg/mL, and endogenous upper limit of quantification was established as 7310 pg/mL. Internal quality controls were included for validation across runs. Concentrations were interpolated from the standard using a four-parameter logistic curve fit, with 1/y^2^ weighting and adjusted for dilution factor.

CSF levels of soluble colony-stimulating factor 1 receptor (sCSF1R) were determined using DuoSet ELISA (R&D Systems) according to manufacturer’s instructions. The CSF samples were diluted at 1:64 and tested in duplicate. The quantitative range of the assay was determined to be 62.5‒8000 pg/mL. CSF endogenous quality control samples and internal quality controls were included for validation across runs. Concentrations were interpolated from the standard using a four-parameter logistic curve fit, with 1/y^2^ weighting and adjusted for dilution factor.

CSF human osteopontin/secreted phosphoprotein 1 (OPN/SPP1) levels were quantified using the human osteopontin Simple Plex assay (ProteinSimple) employed on the Ella platform according to the manufacturer’s instructions, with calibration performed using the in-cartridge factory standard curve. CSF samples were diluted 1:100 in Sample Diluent 13 (ProteinSimple), and the microfluidic platform enabled use of a single well to yield triplicate assay results. The quantitative range of the assay was 1.34‒32,000 pg/mL. Using endogenous quality control CSF samples, the lowest endogenous levels of quantitation was established from parallelism data as 184,861 pg/mL, and the highest endogenous upper limit of quantification was established as 688,491 pg/mL. Concentrations were interpolated from the standard using a five-parameter logistic curve fit, with 1/y^2^ weighting and adjusted for dilution factor.

*Volumetric MRI analyses*

High-resolution 3D T1-weighted images were analysed to quantify brain volumes using SIENAX,^S1^ part of the FMRIB Software Library (FSL) software platform.^S2^ SIENAX starts by extracting brain and skull images from the single whole-head input data. The brain image was then affine registered to MNI152 standard space (using the skull image to determine the registration scaling).^S3^ Next, tissue-type segmentation with partial volume estimation was carried out to calculate estimates of brain volume, grey matter volume, white matter volume, and ventricular volume. Where needed, the ventricle segmentation was manually refined to adjust for the enlarged ventricles observed in CSF1R-ALSP. Ventricle volume included the volume of the lateral ventricle and the third ventricle anatomical structure. Grey matter volume was corrected for potential white matter lesions being segmented as grey matter (due to image contrast) by deducting volume segmented as grey matter within the white matter lesion mask.

Additionally, a transformation to MNI152 space was obtained using FSL FLIRT,^S3^ which implements a linear transformation of the native to MNI152 space that is further refined using nonlinear warping via FSL FNIRT.^S4^ These transformations were used to obtain an estimate of corpus callosum volume through transformation of a corpus callosum mask as defined in MNI152 standard space to the participant's T1-weighted native space image and subsequent masking with the previously obtained white matter tissue mask for this participant. Finally, the corpus callosum thickness was extracted as the average height in the z-direction across the body of the corpus callosum.

T2-weighted FLAIR (fluid-attenuated inversion recovery) images were used to detect and quantify the volume of white matter lesions. Segmentation of white matter lesions was performed using a semi-automated approach due to the high heterogeneity of lesion load in CSF1R-ALSP.

First, candidate white matter hyperintensities (WMHs) were detected using two tools: FSL-BIANCA and FSL-FAST.^S5,S6^ FSL-BIANCA is an automated, supervised method for WMH detection based on the k-NN (k‒nearest neighbour) algorithm. BIANCA classifies the voxels in the image based on their intensity and spatial features, and the output image represents the probability per voxel of being WMH. As training data for this particular type of WMH are not available, an alternative set was used, and the resulting probability maps were thresholded at two thresholds (0.2 and 0.9) and binarised. In addition, FSL-FAST segments a 3D image of the brain into different tissue types (4-class segmentation used: grey matter, white matter, CSF, WMH), while also correcting for spatial intensity variations. The partial volume tissue segmentation for the WMH class was then thresholded (0.99) and binarised (WMH_fast). Second, an initial automated cleaning step was performed by masking out from the WMH masks the subcortical structures segmented with FSL-FIRST.^S7^ Finally, for each scan, the best option was selected from the three available masks (BIANCA 0.2, BIANCA 0.9, or FAST). Importantly, when processing follow-up time points, the same option that had been selected at baseline (BIANCA 0.2, BIANCA 0.9, or FAST) was used, and the manual editing was informed by the previous scan (i.e. editing performed with the previous scan and segmentation open for reference). At baseline, the selection was based on which option would require the least amount of manual editing. BIANCA 0.9 is usually more appropriate for low lesion load scans, BIANCA 0.2 for high lesion load scans, and FAST for cases with extremely high lesion load. After mask selection, any residual false-positive and false-negative voxels were manually edited using FSLeyes.

*Additional clinical assessments*

Motor assessments were conducted at screening/baseline and months 6, 12, 18, 24, 30, and 36/end of treatment (ET) using the Two-Minute Walk Test (2MWT)^S8^ and the Timed Up and Go (TUG) Test.^S9^ 2MWT assesses self-paced walking ability and functional capacity by measuring the distance an individual can walk in 2 minutes. The TUG test is used to evaluate individuals with conditions that can affect ambulation and balance by measuring the time needed to progress from sitting to standing and walking. These motor assessments are widely used in neurological research to assess motor function,^S8,S9^ but the performance of these tests in CSF1R-ALSP has not been previously reported.

Other functional and neuropsychiatric measures included in this study were the Functional Assessment Questionnaire (FAQ; completed by study partners) and the Zarit Burden Interview (ZBI; completed by study partners), which were administered at baseline and months 6, 12, 18, 24, 30, and 36/ET. Although the functional and neuropsychiatric domains assessed by these tools are relevant to CSF1R-ALSP, the measures have not been reported in CSF1R-ALSP research. The FAQ is a 10-item questionnaire that measures instrumental activities of daily living, such as preparing balanced meals and managing personal finances.^S10^ Each function is rated from 0 to 3 (0=normal, 1=has difficulty but can do alone, 2=requires assistant, 3=totally dependent on others to do), and the FAQ score ranges from 0 to 30, with lower scores indicating better function. The ZBI is a caregiver self-report measure that is used to assess the burden of the disease on the primary caregiver.^S11^ The ZBI comprises 22 items, each rated by the study partner using a five-point scale that ranges from 0=never to 4=nearly always. The ZBI total score is the sum of the 22 item scores and ranges from 0 to 88. The FAQ and ZBI were not completed if the participant did not have a study partner who provided informed consent for the study.

**Supplementary Table 1 ILLUMINATE study primary investigators and institutional review boards (IRBs)/independent ethics committees (IECs)**

| **Name** | **Study site** | **IRB/IEC name [approval number]** | **Contact email** |
| --- | --- | --- | --- |
| **Florian Eichler** | Department of Neurology  Massachusetts General Hospital and Harvard Medical School  Boston, MA, USA | Advarra [CR00673910] | feichler@partners.org |
| **Elizabeth Finger** | Western University  London, ON, Canada | Western Research HSREB [reference number: 2025-119172-110267] | elizabeth.finger@lhsc.on.ca |
| **Jeffrey Gelfand** | University of California-San Francisco  San Francisco, CA, USA | University of San Francisco IRB [445094] | jeffrey.gelfand@ucsf.edu |
| **Stuart Isaacson** | Parkinson’s Disease and Movement Disorders Center of Boca Raton  Boca Raton, FL, USA | Advarra [CR00553030] | isaacson_research@parkinsonscenter.org |
| **Wolfgang Köhler** | University of Leipzig Medical Centre  Leipzig, Germany | University of Leipzig Ethics Committee [reference number: 545/21-ek] | wolfgang.koehler@medizin.uni-leipzig.de |
| **Rajeev Kumar** | CenExel RMCR  Englewood, CO, USA | HCA-HealthONE IRB | r.kumar@cenexel.com |
| **David Lynch** | National Hospital for Neurology & Neurosurgery, Queen Square, and UCL Institute of Neurology  London, United Kingdom | Coventry and Warwick Regulatory Ethics Committee [REC Reference - 21/WM/0224] | david.lynch.13@ucl.ac.uk |
| **Jennifer Orthmann-Murphy** | University of Pennsylvania  Philadelphia, PA, USA | Advarra (Central) [CR00668007]  Penn IRB (Local) [confirmation number: edjjgjdf] | jennifer.orthmann-murphy@pennmedicine.upenn.edu |
| **Ludger Schöls** | Tübingen University Hospital  Tübingen, Germany | Ethics Committee of the Medical Faculty of Eberhard Karl University Tübingen [reference number: 325/2022BO2] | ludger.schoels@uni-tuebingen.de |
| **Paulo Sgobbi** | Federal University of São Paulo (UNIFESP)  São Paulo, SP, Brazil | Ao Comite de Etica em Pesquisa em Seres Humanos do Centro Universitario FMABC | paulo@psegtrials.com |
| **Nicole Wolf** | Amsterdam Leukodystrophy Center, Dept. of Child Neurology, Emma Children’s Hospital, Amsterdam UMC, Vrije Universiteit Amsterdam, and Amsterdam Neuroscience  Amsterdam, Netherlands | Medical Ethical Committee University of Groningen (METC UMCG) [research portal number: NL-007707] | n.wolf@amsterdamumc.nl |
| **Zbigniew Wszolek** | Mayo Clinic Florida  Jacksonville, FL, USA | Mayo Clinic IRB [application identification number: 21-006198] | wszolek.zbigniew@mayo.edu |

**Supplementary Table 2 Details of biomarker assay performance**

| **Parameter** | **NfL** | | **GFAP** | | **sTREM2** | **sCSF1R** | **OPN/SPP1** |
| --- | --- | --- | --- | --- | --- | --- | --- |
| **Platform** | Protein Simple Ella SimplePlex | | MSD S-Plex | | R&D systems DuoSet ELISA | R&D systems DuoSet ELISA | Protein Simple Ella SimplePlex |
| **Matrix** | CSF | Serum | CSF | Serum | CSF | CSF | CSF |
| **Minimum required dilution** | 1:2 | 1:2 | 1:32 | 1:8 | 1:5 | 1:64 | 1:100 |
| **Calibration (quantitative range)** | 2.70‒10,290 pg/mL | | 0.51‒781 pg/mL | | 9.96‒6080 pg/mL | 62.5‒8000 pg/mL | 1.34‒32,000 pg/mL |
| **Endogenous LLOQ^a^** | 148 pg/mL | 6.82 pg/mL | 1049 pg/mL | 21.5 pg/mL | 230 pg/mL | 11,539 pg/mL^b^ | 184,861 pg/mL |
| **Endogenous ULOQ^a^** | 14,976 pg/mL | 136 pg/mL | 4305 pg/mL | 110 pg/mL | 7310 pg/mL | 293,341 pg/mL^b^ | 688,491 pg/mL |
| **Intra-assay %CV (buffer QC)** | 3.9±0.3% | | 5.7±3.8% | | 3.8±0.9% | 8.8±3.0% | 9.5±0.4% |
| **Intra-assay %CV (endogenous QC)** | 6.6±0.8% | 8.4±6.6% | 5.0±0.1% | 6.6±1.8% | 7.2±3.7% | 12.2±2.8% | 7.3±0.3% |
| **Inter-assay %CV (buffer QC)** | 8.4±0.9% | | 9.9±5.2% | | 9.4±3.3% | 10.8±2.3% | 14.3±2.3% |
| **Inter-assay %CV (endogenous QC)** | 7.7±0.9% | 11.2±7.8% | 13.0±2.8% | 11.0±2.7% | 13.7±1.6% | 12.7±1.1% | 12.6±1.3% |
| **Sample testing** | Duplicate (2 wells,  6 GNR - 6 replicates) | | Duplicate (2 wells) | | Duplicate (2 wells) | Duplicate (2 wells) | Single (1 well,  3 GNR - triplicate) |
| **Study sample QC acceptance criteria** | <20% CV for at least 3 out of 6 replicates (2 wells) | | <20% CV for duplicate  (2 wells) | | <20% CV for duplicate  (2 wells) | <20% CV for duplicate  (2 wells) | <20% CV for triplicate  (1 well) |

Calculations based on validation summary data from assay-specific method validation reports.

^a^Established from parallelism assessment.

^b^Established in-study, as parallelism could not be evaluated with samples available during validation.

CSF=cerebrospinal fluid; CV=coefficient of variance; GFAP=glial fibrillary acidic protein; GNR=glass nanoreactor; LLOQ=lower level of quantification; NfL=neurofilament light chain; OPN/SPP1=osteopontin/secreted phosphoprotein 1; QC=quality control; sCSF1R=soluble colony stimulating factor 1 receptor; sTREM2=soluble triggering receptor on myeloid cells 2; ULOQ=upper level of quantification.

**Supplementary Table 3 *CSF1R* variant classification and evidence summary**

|  | **Amino acid change** | **Nucleic acid change** | **Exon** | **Mutation within TKD** | **Type** | **No. of individuals** | **Clin variant classification** | **Presence in population databases** | **Functional studies** | **Literature references (PMID)** |
| --- | --- | --- | --- | --- | --- | --- | --- | --- | --- | --- |
| 1 | p.C224S | c.670T>A | 6 | No | Missense | 1 | Variant of uncertain significance | No frequency | Output from modelling did not meet the statistical confidence thresholds | Not reported |
| 2 | p.R579W | c.1735C>T | 13 | Yes | Missense | 2 | Likely pathogenic | No frequency | No data | 38465843, 39280886 |
| 3 | p.G589E | c.1766G>A | 13 | Yes | Missense | 2 | Pathogenic | No frequency | Expected to disrupt CSF1R protein function | 27680516, 34652888, 39280886, 36980326, 39805964, 31872055, 35022934 |
| 4 | p.A629S | c.1885G>T | 14 | Yes | Missense | 1 | Variant of uncertain significance | No frequency | No data | Not reported |
| 5 | p.Q642* | c.1924C>T | 14 | Yes | Missense | 2 | Pathogenic | No frequency | It is expected to result in an absent or disrupted protein product | 37292133, 36559271, 34329526 |
| 6 | p.C653Y | c.1958G>A | 14 | Yes | Missense | 1 | Pathogenic | No frequency | Disrupts a conserved cysteine residue in the TKD, likely impairing autophosphorylation and downstream signalling. | 33553700, 27608278, 23038421, 25383640 |
| 7 | p.C666R | c.1996C>T | 15 | Yes | Missense | 1 | Likely pathogenic | No frequency | No data | 37292133, 36559271, 31840744 |
| 8 | p.G669D | c.2006G>A | 15 | Yes | Missense | 1 | Variant of uncertain significance | No frequency | Variant is likely to be disruptive | 40489910 |
| 9 | p.M766T | c.2297T>C | 16 | Yes | Missense | 1 | Variant of uncertain significance | No frequency | No data | 38465843, 27680516, 22197934, 22197934, 23787135, 27633805, 30528841, 23408870 |
| 10 | p.A770P | c.2308G>C | 16 | Yes | Missense | 1 | Pathogenic | No frequency | No data | 34541732, 27680516, 22197934, 22843259, 23787135, 30115677 |
| 11 | p.R777W | c.2329C>T | 18 | Yes | Missense | 1 | Pathogenic | No frequency | Output from this modeling did not meet the statistical confidence thresholds | 39280886, 23649896, 27680516, 31520500, 24390523, 30268725, 35119108 |
| 12 | p.A781_N783del | c.2342_2350DEL | 18 | Yes | Frameshift | 3 | Pathogenic | No frequency | Expected to disrupt CSF1R protein function | 37292133, 33350588, 34908483 |
| 13 | p.A781V | c.2342C>T | 18 | Yes | Missense | 1 | Pathogenic | No frequency | Output from this modeling did not meet the statistical confidence thresholds | 38465843, 35389179, 27680516, 34541732, 28243630, 31827782, 30115677, 30217938, 37253125, |
| 14 | p.R782H | c.2345G>A | 18 | Yes | Missense | 2 | Pathogenic | No frequency | Functional studies demonstrate a damaging effect, revealing loss of autophosphorylation of selected tyrosine residues in the kinase domain | 27680516, 31520500, 36559271, 34541732, 36980326, 38805875, 24390523, 25563800, 27619214, 33866445, 22503135, 26141825 |
| 15 | p.L786S | c.2357T>C | 18 | Yes | Missense | 1 | Variant of uncertain significance | No frequency | No data | 40489910, 37292133, 36559271, 34329526 |
| 16 | p.A792D | c.2375C>A | 18 | Yes | Missense | 1 | Likely pathogenic | No frequency | No data | 27680516, 32276111, 37292133, 36559271, 33528831, 35856733 |
| 17 | p.I794T | c.2381T>C | 18 | Yes | Missense | 7 | Pathogenic | 0.0009 (genomAD) | Output from this modeling did not meet the statistical confidence thresholds | 28059798, 38465843, 34422984, 35389179, 36943150, 27680516, 39280886, 33553700, 27608278, 31827782, 36559271, 34541732, 31520500, 28243630, 28334938, 32705467, 24336230, 38805875, 27490250, 24390523, 36380532, 30115677, 24198292, 34837774, 31705326, 26141177, 29983329, 35332077, 35532660, 35532660, 33999983, 35980505, 36207627, 15365727, 29044417, 25390884, 18794495, 30968732, 23052599 |
| 18 | p.G798R | c.2392G>A | 18 | Yes | Missense | 1 | Likely pathogenic | No frequency | No data | 39280886, 28122429 |
| 19 | p.G798R | c.2392G>C | 18 | Yes | Missense | 1 | Likely pathogenic | No frequency | Expected to disrupt CSF1R protein function | 31520500 |
| 20 |  | c.2442+1G>A | 18/19 | Yes | Intronic, splice donor | 2 | Likely pathogenic | No frequency | No data | 38465843, 35389179, 27680516, 37292133, 36559271, 31520500, 25935893, 28243630, 34329526, 31418856 |
| 21 |  | c.2442+2T>C | 18/19 | Yes | Intronic, splice donor | 1 | Likely pathogenic | No frequency | Canonical splice site variant expected to result in aberrant splicing, although in the absence of functional evidence the actual effect of this sequence change is unknown | 40489910, 27423618 |
| 22 | p.E825Q | c.2473G>C | 19 | Yes | Missense | 1 | Variant of uncertain significance | No frequency | In silico analysis indicates that this missense variant does not alter protein structure/function | 29509319, 25935893, 28243630, 29700822, 38465843 |
| 23 | p.S836N | c.2507G>A | 19 | Yes | Missense | 2 | Variant of uncertain significance | No frequency | No data | Not reported |
| 24 | p.L845F | c.2533C>T | 19 | Yes | Missense | 1 | Variant of uncertain significance | No frequency | In silico analysis supports that this missense variant has a deleterious effect on protein structure/function | Not reported |
| 25 | p.E847K | c.2539G>A | 19 | Yes | Missense | 1 | Pathogenic | No frequency | Expected to disrupt CSF1R protein function | 26401554, 30853829 |
| 26 | p.F849I | c.2545T>A |  | Yes | Missense | 1 | Likely pathogenic | No frequency | Five of five in-silico tools predict a damaging effect of the variant on protein function. | 36943150 |
| 27 | p.N854K | c.2562T>A | 20 | Yes | Missense | 1 | Variant of uncertain significance | 0.00002 (genomAD), 0.00002 (ExAC), 0.00005 (TOPMed) | In silico analysis, which includes protein predictors and evolutionary conservation, supports that this variant does not alter protein structure/function | 38465843, 31827782, 31093799, 25311247, 26756564, 32453488 |
| 28 | p.N854K | c.2563C>A | 20 | Yes | Missense | 1 | Likely pathogenic | No frequency | The prevalence of the variant in affected individuals is significantly increased compared to the prevalence in controls | 38465843, 27163664 |
| 29 | p.V869G | c.2606T>G | 20 | Yes | Missense | 1 | Variant of uncertain significance | No frequency | Expected to be pathogenic, be pathogenic, at this time its clinical significance is uncertain due to the absence of conclusive functional and genetic evidence | 36943150, 30217938, 27163664 |
| 30 | p.G872R | c.2614 G>A | 20 | Yes | Missense | 1 | Variant of uncertain significance | No frequency | Supports that this missense variant has a deleterious effect on protein structure/function | 35812083 |
| 31 | p.M875T | c.2625G>A | 20 | Yes | Missense | 1 | Pathogenic | No frequency | No data | 37292133, 36559271, 34329526, 30901701, 37434390, 33590562, 34145637, 34145638, 35044689 |
| 32 | p.Q877* | c.2629C>T | 20 | Yes | Missense | 1 | Likely pathogenic | No frequency | No data | 24198292, 30217938 |
| 33 | p.P878T | c.2632C>A | 20 | Yes | Missense | 1 | Variant of uncertain significance | No frequency | Output from modeling did not meet the statistical confidence thresholds | 22197934; 27680516 |
| 34 | p.Y886SfsX56 | c.2656_2657insC | 20 | Yes | Frameshift | 2 | Pathogenic | No frequency | No data | 35940158 |
| 35 | p.M889L | c.2665A>C | 20 | Yes | Missense | 1 | Likely pathogenic | No frequency | No data | 37292133 |
| 36 | p.W893R | c.2677T>C | 20 | Yes | Missense | 1 | Variant of uncertain significance | No frequency | Supports a deleterious effect | 37292133; 36559271; 34329526 |
| 37 | p.I906T | c.2717T>C | 20 | Yes | Missense | 1 | Variant of uncertain significance | No frequency | Output from modeling did not meet the statistical confidence thresholds | 27680516; 31827782 |

All reported cases met the study inclusion and exclusion criteria. All participants, aged at least 18 years, were required to have a documented *CSF1R* variant and brain MRI findings consistent with CSF1R-ALSP. Symptomatic patients must have experienced clinical progression of CSF1R-ALSP within the past year and have a baseline MoCA score of at least 12 and more than two signs or symptoms on a neurological evaluation in any of the following categories: cognitive impairment, psychiatric problems, pyramidal signs, extrapyramidal features, or epilepsy. Prodromal cases were required to have fewer than two CSF1R-ALSP–related signs or symptoms. Patients with variants classified as variants of uncertain significance were considered for inclusion by experts experienced in CSF1R-ALSP; only cases with a clear clinical and radiological presentation were included, especially if supported by family history and literature. MoCA=Montreal Cognitive Assessment; PMID=PubMed ID; TKD=tyrosine kinase domain.

**Supplementary Table 4 Summary of fluid biomarker levels by visit**

| **Biomarker,^a^ pg/mL** | **Prodromal (*n*=19)** | | | **Symptomatic-no HSCT (*n*=23)** | | | **Symptomatic+HSCT (*n*=11)** | | |
| --- | --- | --- | --- | --- | --- | --- | --- | --- | --- |
|  | ***n*** | **Mean** (**SD)** | **Median (min, max)** | ***n*** | **Mean** (**SD)** | **Median (min, max)** | ***n*** | **Mean** (**SD)** | **Median (min, max)** |
| **NfL (serum)** |  |  |  |  |  |  |  |  |  |
| Baseline | 17 | 27.0 (30.9) | 22.0 (7.1, 140.0) | 22 | 117.4 (69.7) | 121.5 (10.9, 265.0) | 11 | 15.6 (4.73) | 17.4 (7.3, 21.7) |
| Month 6 | 14 | 17.6 (6.5) | 17.4 (7.0, 27.0) | 12 | 123.6 (88.3) | 111.0 (17.2, 334.0) | 7 | 16.0 (9.8) | 12.9 (6.7, 35.2) |
| Month 12 | 17 | 19.4 (9.1) | 16.2 (9.0, 33.5) | 7 | 143.3 (109.1) | 139.0 (10.6, 344.0) | 9 | 18.5 (7.9) | 16.4 (7.4, 29.2) |
| Month 18 | 6 | 21.7 (5.1) | 22.6 (13.9, 26.9) | 1 | 86.3 (NA) | NA | 3 | 12.2 (2.4) | 11.1 (10.5, 15.0) |
| Month 24 | 9 | 16.9 (10.1) | 12.1 (6.4, 36.0) | 3 | 149.9 (95.1) | 188.0 (41.6, 220.0) | 9 | 12.7 (4.0) | 12.7 (6.6, 18.6) |
| Month 30 | 4 | 19.6 (7.9) | 20.2 (11.7, 26.4) | 2 | 96.0 (55.2) | 96.0 (57.0, 135.0) | 5 | 14.4 (6.4) | 12.4 (6.9, 23.8) |
| Month 36 | 1 | 28.9 (NA) | NA | 0 | NA | NA | 0 | NA | NA |
| **NfL (CSF)** |  |  |  |  |  |  |  |  |  |
| Baseline | 4 | 704.0 (324.0) | 630.5 (395.0, 1160.0) | 14 | 7989.9 (6483.5) | 6090.0 (271.0, 22,799.0) | 4 | 1166.8 (659.2) | 927.0 (682.0, 2131.0) |
| Month 6 |  | NA | NA | 2 | 12,035.0 (7286.0) | 12,035.0 (6883.0, 17,187.0) |  | NA | NA |
| Month 12 | 1 | 835.0 (NA) | NA | 2 | 5937.5 (3687.6) | 5937.5 (3330.0, 8545.0) | 2 | 909.0 (315.4) | 909.0 (686.0, 1132.0) |
| Month 24 | 0 | NA | NA | 0 | NA | NA | 1 | 662.0 (NA) | NA |
| **GFAP (serum)** |  |  |  |  |  |  |  |  |  |
| Baseline | 18 | 88.7 (59.5) | 72.5 (46.0, 291.0) | 23 | 175.2 (136.4) | 159.0 (42.7, 702.0) | 11 | 88.5 (45.4) | 73.8 (44.4, 201.0) |
| Month 6 | 14 | 78.9 (51.0) | 69.2 (34.3, 232.0) | 12 | 226.0 (232.0) | 154.0 (74.9, 929.0) | 7 | 72.9 (20.4) | 67.5 (42.4, 106.0) |
| Month 12 | 17 | 92.0 (62.5) | 85.3 (31.4, 313.0) | 7 | 284.1 (276.9) | 203.0 (110.0, 903.0) | 9 | 108.0 (66.6) | 105.0 (43.2, 264.0) |
| Month 18 | 7 | 99.4 (61.1) | 79.0 (44.6, 228.0) | 1 | 145.0 (NA) | NA | 3 | 102.2 (29.6) | 89.4 (81.1, 136.0) |
| Month 24 | 11 | 82.0 (30.5) | 77.9 (44.6, 148.0) | 3 | 289.0 (110.3) | 326.0 (165.0, 376.0) | 9 | 96.9 (54.1) | 93.7 (26.6, 196.0) |
| Month 30 | 5 | 74.9 (13.6) | 76.0 (56.2, 93.2) | 2 | 261.0 (32.5) | 261.0 (238.0, 284.0) | 5 | 75.5 (28.4) | 65.5 (43.7, 113.0) |
| Month 36 | 3 | 51.7 (9.8) | 53.5 (41.2, 60.5) | 0 | NA | NA | 0 | NA | NA |
| **GFAP (CSF)** |  |  |  |  |  |  |  |  |  |
| Baseline | 4 | 5831.5 (2078.7) | 5507.5 (4016.0, 8295.0) | 14 | 10,828.9 (5795.5) | 10,269.5 (1601.0, 22,780.0) | 4 | 6469.5 (3202.2) | 6249.0 (3562.0, 9818.0) |
| Month 6 |  | NA | NA | 2 | 15,945.5 (258.1) | 15,945.5 (15,763.0, 16,128.0) |  | NA | NA |
| Month 12 | 1 | 3951.0 (NA) | NA | 2 | 9294.5 (3937.9) | 9294.5 (6510.0, 12,079.0) | 2 | 9628.5 (1519.6) | 9628.5 (8554.0, 10,703.0) |
| Month 24 | 0 | NA | NA | 0 | NA | NA | 1 | 8471.0 (NA) | NA |
| **sCSF1R (CSF)** |  |  |  |  |  |  |  |  |  |
| Baseline | 4 | 25,592.0 (9844.7) | 23,544.0 (15,938.0, 39,342.0) | 14 | 22,002.1 (9172.2) | 20,766.5 (8552.0, 39,888.0) | 4 | 65,368.8 (12,196.2) | 63,921.0 (52,260.0, 81,373.0) |
| Month 6 |  | NA | NA | 2 | 28,962.5 (16,056.3) | 28,962.5 (17,609.0, 40,316.0) |  | NA | NA |
| Month 12 | 1 | 23,555.0 (NA) | NA | 2 | 22,128.0 (12,436.6) | 22,128.0 (13,334.0, 30,922.0) | 2 | 96,394.0 (46,404.6) | 96,394.0 (63,581.0, 12,9207.0) |
| Month 24 | 0 | NA | NA | 0 | NA | NA | 1 | 61,675.0 (NA) | NA |
| **sTREM2 (CSF)** |  |  |  |  |  |  |  |  |  |
| Baseline | 4 | 1964.3 (715.9) | 2102.5 (1001.0, 2651.0) | 14 | 2577.1 (1279.7) | 2220.5 (924.0, 4602.0) | 4 | 2869.8 (1000.6) | 2918.5 (1632.0, 4010.0) |
| Month 6 |  | NA | NA | 2 | 3777.0 (2525.8) | 3777.0 (1991.0, 5563.0) |  | NA | NA |
| Month 12 | 1 | 2543.0 (NA) | NA | 2 | 1733.0 (188.1) | 1733.0 (1600.0, 1866.0) | 2 | 4229.5 (863.4) | 4229.5 (3619.0, 4840.0) |
| Month 24 | 0 | NA | NA | 0 | NA | NA | 1 | 2262.0 (NA) | NA |
| **OPN/SPP1 (CSF)** |  |  |  |  |  |  |  |  |  |
| Baseline | 4 | 509,343.8 (109,784.0) | 534,087.5 (365,437.0, 603,763.0) | 14 | 507,206.1 (194,005.5) | 495,522.0 (226,394.0, 968,564.0) | 4 | 305,085.3 (128,814.1) | 293,994.5 (159,717.0, 472,635.0) |
| Month 6 |  | NA | NA | 2 | 806,506.5 (475,404.2) | 806,506.5 (470,345.0, 1,142,668.0) |  | NA | NA |
| Month 12 | 1 | 561,435.0 (NA) | NA | 2 | 450,655.0 (102,063.8) | 450,655.0 (378,485.0, 522,825.0) | 2 | 292,645.0 (4348.7) | 292,645.0 (289,570.0, 295,720.0) |
| Month 24 | 0 | NA | NA | 0 | NA | NA | 1 | 424,936.0 (NA) | NA |

^a^For comparison, normative values are presented in **Supplementary Table 5**.

CSF=cerebrospinal fluid; GFAP=glial fibrillary acidic protein; HSCT=haematopoietic stem cell transplant; NA=not applicable; NfL=neurofilament light chain; OPN/SPP1=osteopontin/secreted phosphoprotein 1; sCSF1R=soluble colony stimulating factor 1 receptor; SD=standard deviation; sTREM2=soluble triggering receptor on myeloid cells 2.

**Supplementary Table 5 Normative biomarker levels^a^ from healthy volunteers from the first-in-human single-/multiple-ascending dose study of iluzanebart^S12^**

| **Biomarker, pg/mL** | ***n*** | **Concentration, mean (SD)** |
| --- | --- | --- |
| NfL (serum) | 67^a^ | 10.1 (5.5) |
| NfL (CSF)^b^ | 24^a^ | 801 (1605) |
| GFAP (serum) | 69 | 44.2 (19.9) |
| GFAP (CSF) | 24^a^ | 2792 (1115) |
| sCSF1R (CSF) | 51 | 117,120 (34,333) |
| sTREM2 (CSF) | 51 | 3273 (1406) |
| OPN/SPP1 (CSF) | 51 | 408,533 (170,471) |

^a^Analytes were analysed at different times and within different cohorts. Of the cohort of 69 participants from whom serum samples were reported in the VGL101-01.101 phase 1 study, 2 values for serum NfL could not be reported: 1 value was below the limit of quantitation, and 1 was haemolysed (resulting in *n*=67). Of the cohort of 51 participants from whom CSF samples were reported in the VGL101-01.101 phase 1 study, data for CSF NfL and GFAP were available from only 24 participants.

^b^One individual had a CSF NfL concentration that was 4.5 SDs higher than the mean; a sensitivity analysis omitting this individual resulted in mean (SD) of 477 (255) pg/mL.

CSF=cerebrospinal fluid; GFAP=glial fibrillary acidic protein; NfL=neurofilament light chain; OPN/SPP1=osteopontin/secreted phosphoprotein 1; sCSF1R=soluble colony stimulating factor 1 receptor; SD=standard deviation; sTREM2=soluble triggering receptor on myeloid cells 2.

## **Supplementary Table 6 Statistical comparisons between normative biomarker levels from healthy volunteers with baseline levels in prodromal patients, symptomatic-patients with no HSCT, and symptomatic-patients with HSCT**

| **Biomarker, adjusted P values** | **HV vs Prodromal** | **HV vs symptomatic-no HSCT** | **HV vs symptomatic+HSCT** | **Prodromal vs symptomatic-no HSCT** | **Prodromal vs symptomatic+HSCT** | **Symptomatic-no HSCT vs symptomatic+HSCT** |
| --- | --- | --- | --- | --- | --- | --- |
| Serum NfL | 0.1964 (n=67 vs 17) | **<0.0001** (n=67 vs 22) | **0.02** (n=67 vs 11) | **<0.001** (n=17 vs 22) | 0.5898 (n=17 vs 11) | **<0.0001** (n=22 vs 11) |
| CSF NfL | 0.9999 (n=24 vs 4) | **0.0065** (n=24 vs 14) | 0.958 (n=24 vs 4) | **0.0061** (n=4 vs 14) | 0.7493 (n=4 vs 4) | **0.0097** (n=14 vs 4) |
| Serum GFAP | **0.0328** (n=69 vs 18) | **0.0008** (n=69 vs 23) | **0.0463** (n=69 vs 11) | 0.0585 (n=18 vs 23) | >0.9999 (n=18 vs 11) | 0.0571 (n=23 vs 11) |
| CSF GFAP | 0.2211 (n=24 vs 4) | **0.0009** (n=24 vs 14) | 0.3474 (n=24 vs 4) | 0.0917 (n=4 vs 14) | 0.9993 (n=4 vs 4) | 0.3506 (n=14 vs 4) |
| CSF sCSF1R | **<0.0001** (n=51 vs 4) | **<0.0001** (n=51 vs 14) | **0.0009** (n=51 vs 4) | 0.9777 (n=4 vs 14) | **0.0113** (n=4 vs 4) | **0.0118** (n=14 vs 4) |
| CSF sTREM2 | 0.1037 (n=51 vs 4) | 0.415 (n=51 vs 14) | 0.9561 (n=51 vs 4) | 0.7618 (n=4 vs 14) | 0.6333 (n=4 vs 4) | 0.9953 (n=14 vs 4) |
| CSF OPN/SPP1 | 0.5353 (n=51 vs 4) | 0.4404 (n=51 vs 14) | 0.6226 (n=51 vs 4) | >0.9999 (n=4 vs 14) | 0.2224 (n=4 vs 4) | 0.1985 (n=14 vs 4) |

Pairwise post hoc comparisons were conducted using two‑sample t-tests with Welch’s correction to account for unequal variances. Adjustment for multiple comparisons within each analyte was performed using the Dunnett T3 procedure. CSF=cerebrospinal fluid; GFAP=glial fibrillary acidic protein; HSCT=haematopoietic stem cell transplant; HV=healthy volunteer; NfL=neurofilament light chain; OPN/SPP1=osteopontin/secreted phosphoprotein 1; sCSF1R=soluble colony stimulating factor 1 receptor; sTREM2=soluble triggering receptor on myeloid cells 2.

**Supplementary Table 7 Summary of MRI outcomes by visit**

| **MRI outcome** | **Prodromal (*n*=19)** | | | **Symptomatic-no HSCT (*n*=23)** | | | **Symptomatic+HSCT (*n*=11)** | | |
| --- | --- | --- | --- | --- | --- | --- | --- | --- | --- |
|  | ***n*** | **Mean** (**SD)** | **Median (min, max)** | ***n*** | **Mean** (**SD)** | **Median (min, max)** | ***n*** | **Mean** (**SD)** | **Median (min, max)** |
| **Ventricle volume, mL** |  |  |  |  |  |  |  |  |  |
| Baseline | 19 | 30.0 (15.8) | 24.1 (8.7, 70.3) | 23 | 67.4 (27.0) | 61.5 (26.3, 139.8) | 11 | 117.5 (38.5) | 102.1 (66.2, 199.4) |
| Month 6 | 18 | 31.1 (16.3) | 26.3 (8.9, 71.2) | 14 | 70.3 (27.1) | 65.2 (26.4, 118.3) | 10 | 122.8 (38.4) | 113.0 (79.8, 200.8) |
| Month 12 | 18 | 31.6 (16.7) | 26.5 (9.4, 72.8) | 7 | 83.0 (39.6) | 74.2 (37.8, 160.8) | 9 | 116.3 (37.7) | 94.8 (88.1, 198.4) |
| Month 18 | 17 | 32.4 (17.3) | 27.9 (8.7, 72.3) | 2 | 124.1 (64.7) | 124.1 (78.3, 169.8) | 8 | 120.3 (41.5) | 99.7 (82.7, 201.2) |
| Month 24 | 11 | 28.8 (14.1) | 25.4 (9.0, 54.9) | 1 | 81.7 (NA) | NA | 9 | 118.1 (40.6) | 95.5 (75.2, 198.0) |
| Month 30 | 6 | 29.2 (15.7) | 24.6 (15.0, 59.2) | 1 | 86.7 (NA) | NA | 4 | 149.9 (43.1) | 154.1 (94.3, 197.2) |
| Month 36 | 3 | 22.3 (8.3) | 22.3 (13.9, 30.5) | 0 | NA | NA | 0 | NA | NA |
| **GM volume, mL** |  |  |  |  |  |  |  |  |  |
| Baseline | 19 | 582.1 (67.0) | 570.3 (470.5, 716.7) | 23 | 528.3 (55.1) | 529.9 (418.3, 672.6) | 11 | 491.7 (48.0) | 484.5 (423.7, 555.5) |
| Month 6 | 18 | 583.7 (69.6) | 582.7 (471.3, 720.1) | 13 | 518.8 (68.2) | 505.1 (396.8, 688.5) | 10 | 485.3 (59.2) | 480.4 (398.6, 559.4) |
| Month 12 | 18 | 580.5 (68.3) | 577.4 (459.0, 700.9) | 7 | 505.0 (52.5) | 494.0 (445.3, 592.1) | 9 | 477.7 (66.8) | 470.1 (350.7, 551.6) |
| Month 18 | 17 | 580.1 (69.7) | 576.3 (466.7, 713.7) | 2 | 456.5 (41.5) | 456.5 (427.1, 485.8) | 8 | 477.0 (61.8) | 493.1 (382.6, 546.7) |
| Month 24 | 11 | 567.2 (72.1) | 566.4 (466.1, 713.2) | 1 | 474.4 (NA) | NA | 9 | 489.8 (60.6) | 484.5 (406.2, 572.1) |
| Month 30 | 6 | 587.8 (78.1) | 583.9 (483.2, 705.5) | 1 | 454.7 (NA) | NA | 4 | 532.0 (22.6) | 531.4 (506.1, 559.1) |
| Month 36 | 3 | 565.0 (85.6) | 608.0 (466.4, 620.7) | 0 | NA | NA | 0 | NA | NA |
| **Corpus callosum volume, mL** |  |  |  |  |  |  |  |  |  |
| Baseline | 19 | 31.6 (3.4) | 30.9 (26.2, 39.1) | 23 | 25.8 (5.9) | 24.8 (16.3, 44.5) | 11 | 19.7 (5.5) | 18.8 (11.2, 28.7) |
| Month 6 | 18 | 31.6 (3.4) | 31.4 (25.8, 38.5) | 14 | 25.8 (6.5) | 24.9 (17.0, 44.2) | 10 | 18.7 (5.5) | 18.4 (9.7, 26.9) |
| Month 12 | 18 | 31.6 (3.3) | 31.4 (25.7, 38.6) | 7 | 22.9 (6.0) | 25.1 (13.5, 29.5) | 9 | 18.6 (6.7) | 19.9 (4.2, 28.6) |
| Month 18 | 17 | 31.4 (3.6) | 31.1 (25.1, 38.3) | 2 | 18.3 (9.2) | 18.3 (11.8, 24.8) | 8 | 20.9 (9.7) | 19.9 (10.9, 41.7) |
| Month 24 | 11 | 31.6 (3.3) | 31.6 (25.9, 37.8) | 1 | 24.7 (NA) | NA | 9 | 18.1 (6.3) | 18.9 (4.3, 25.5) |
| Month 30 | 6 | 31.6 (1.8) | 31.6 (29.3, 33.6) | 1 | 24.8 (NA) | NA | 4 | 17.1 (4.4) | 17.3 (11.9, 21.7) |
| Month 36 | 3 | 30.0 (0.6) | 30.0 (29.4, 30.6) | 0 | NA | NA | 0 | NA | NA |
| **Corpus callosum thickness, mm** |  |  |  |  |  |  |  |  |  |
| Baseline | 19 | 6.5 (0.7) | 6.4 (4.9, 8.0) | 23 | 5.0 (1.0) | 4.7 (3.8, 7.2) | 11 | 3.6 (0.7) | 3.7 (1.9, 4.5) |
| Month 6 | 18 | 6.5 (0.8) | 6.4 (4.9, 8.1) | 14 | 5.1 (0.9) | 5.1 (4.2, 7.2) | 10 | 3.4 (0.9) | 3.7 (1.1, 4.5) |
| Month 12 | 18 | 6.4 (0.7) | 6.4 (5.1, 7.9) | 7 | 4.6 (1.2) | 4.8 (2.2, 5.9) | 9 | 3.5 (0.9) | 3.6 (1.6, 4.8) |
| Month 18 | 17 | 6.4 (0.7) | 6.2 (5.2, 7.9) | 2 | 2.8 (2.1) | 2.8 (1.3, 4.3) | 8 | 3.7 (1.3) | 3.9 (1.1, 5.1) |
| Month 24 | 11 | 6.6 (0.7) | 6.4 (5.8, 8.1) | 1 | 4.7 (NA) | NA | 9 | 3.6 (0.9) | 3.6 (1.6, 5.0) |
| Month 30 | 6 | 6.4 (0.5) | 6.4 (5.8, 7.1) | 1 | 4.6 (NA) | NA | 4 | 3.4 (1.6) | 4.0 (1.2, 4.5) |
| Month 36 | 3 | 6.6 (0.3) | 6.4 (6.3, 6.9) | 0 | NA | NA | 0 | NA | NA |
| **Whole brain volume, mL** |  |  |  |  |  |  |  |  |  |
| Baseline | 19 | 1146.9 (124.0) | 1159.3 (898.2, 1350.0) | 23 | 1054.0 (113.7) | 1042.1 (884.4, 1408.9) | 11 | 1016.6 (88.7) | 1033.0 (867.6, 1163.2) |
| Month 6 | 18 | 1145.4 (129.0) | 1156.7 (889.9, 1336.4) | 14 | 1057.5 (142.2) | 1049.6 (812.6, 1422.5) | 10 | 1013.1 (98.4) | 1046.8 (854.0, 1157.5) |
| Month 12 | 18 | 1141.1 (130.4) | 1151.2 (883.8, 1343.7) | 7 | 1043.9 (90.2) | 1036.1 (930.4, 1162.5) | 9 | 1017.9 (102.5) | 1047.4 (820.7, 1181.7) |
| Month 18 | 17 | 1139.9 (131.4) | 1146.6 (882.3, 1330.4) | 2 | 985.2 (62.3) | 985.2 (941.1, 1029.2) | 8 | 1017.6 (94.7) | 1019.0 (831.3, 1151.3) |
| Month 24 | 11 | 1126.0 (139.7) | 1146.8 (882.8, 1324.3) | 1 | 1019.1 (NA) | NA | 9 | 1006.4 (92.5) | 1024.9 (826.9, 1154.4) |
| Month 30 | 6 | 1138.8 (129.8) | 1149.7 (916.7, 1304.8) | 1 | 1007.8 (NA) | NA | 4 | 1064.4 (55.6) | 1058.3 (1006.3, 1134.5) |
| Month 36 | 3 | 1092.5 (164.7) | 1178.9 (902.6, 1196.1) | 0 | NA | NA | 0 | NA | NA |
| **WM lesions volume, mL** |  |  |  |  |  |  |  |  |  |
| Baseline | 19 | 9.8 (9.5) | 5.2 (2.2, 36.2) | 23 | 33.1 (23.4) | 25.5 (2.2, 86.6) | 11 | 42.9 (17.1) | 50.1 (18.5, 67.8) |
| Month 6 | 18 | 8.5 (7.2) | 5.7 (2.5, 27.2) | 13 | 39.8 (32.0) | 28.5 (2.6, 96.7) | 10 | 38.8 (17.6) | 36.8 (16.2, 69.5) |
| Month 12 | 18 | 8.7 (7.4) | 5.0 (2.8, 26.6) | 7 | 50.6 (36.9) | 38.8 (2.2, 100.0) | 9 | 35.1 (17.7) | 26.7 (15.0, 69.6) |
| Month 18 | 17 | 8.6 (7.1) | 4.8 (2.6, 27.5) | 2 | 76.1 (33.6) | 76.1 (52.3, 99.8) | 8 | 34.5 (18.2) | 31.1 (15.2, 69.5) |
| Month 24 | 11 | 7.7 (5.5) | 5.0 (2.5, 20.1) | 1 | 103.9 (NA) | NA | 9 | 33.4 (16.7) | 28.4 (13.6, 66.7) |
| Month 30 | 6 | 9.1 (7.7) | 6.4 (3.5, 24.0) | 1 | 107.5 (NA) | NA | 4 | 25.4 (15.9) | 21.8 (11.6, 46.2) |
| Month 36 | 3 | 5.4 (2.0) | 4.9 (3.6, 7.5) | 0 | NA | NA | 0 | NA | NA |
| **Sundal MRI severity score** |  |  |  |  |  |  |  |  |  |
| Baseline | 19 | 4.0 (4.4) | 4.3 (0.0, 17.0) | 23 | 18.4 (8.9) | 20.8 (1.0, 30.5) | 11 | 26.3 (6.0) | 27.5 (17.5, 34.5) |
| Month 6 | 18 | 4.1 (4.4) | 2.9 (0.0, 17.0) | 14 | 19.1 (10.4) | 22.9 (1.8, 31.3) | 10 | 25.4 (6.0) | 24.6 (14.5, 34.0) |
| Month 12 | 18 | 4.3 (4.6) | 2.9 (0.0, 18.8) | 7 | 22.6 (10.5) | 24.0 (1.5, 36.0) | 9 | 26.0 (4.3) | 25.8 (18.3, 32.5) |
| Month 18 | 17 | 4.8 (5.5) | 2.5 (0.0, 22.5) | 3 | 28.5 (4.8) | 26.3 (25.3, 34.0) | 8 | 25.2 (3.6) | 24.1 (21.5, 31.5) |
| Month 24 | 11 | 3.1 (2.5) | 2.8 (0.0, 9.8) | 2 | 31.1 (6.9) | 31.1 (26.3, 36.0) | 9 | 25.2 (4.1) | 23.5 (20.0, 31.0) |
| Month 30 | 6 | 4.2 (3.2) | 4.4 (0.5, 9.3) | 2 | 34.5 (5.0) | 34.5 (31.0, 38.0) | 4 | 24.2 (2.3) | 23.9 (21.8, 27.3) |
| Month 36 | 3 | 3.3 (2.2) | 3.8 (1.0, 5.3) | 0 | NA | NA | 0 | NA | NA |

GM=grey matter; HSCT=haematopoietic stem cell transplant; MRI=magnetic resonance imaging; NA=not applicable; SD=standard deviation; WM=white matter.

**Supplementary Table 8 Ventricle volume across neurodegenerative diseases**

| **Mean (SD)** | **CSF1R-ALSP (this study)** | | **From Genovese, et al.^30^** | | **From Nestor, et al.^31^** | | |
| --- | --- | --- | --- | --- | --- | --- | --- |
|  | **Prodromal**  **(*n*=19)** | **Symptomatic- no HSCT**  **(*n*=23)** | **Multiple sclerosis**  **(*n*=1341)** | **Progressive multiple sclerosis**  **(*n*=252)** | **Normal elderly control**  **(*n*=152)** | **Mild cognitive impairment**  **(*n*=246)** | **Alzheimer’s disease**  **(*n*=104)** |
| **Ventricle volume, mL** | 30.0 (15.8) | 67.4 (27.0) | 44.9 (19.9) | 56.5 (23.9) | 38.3 (19.1) | 45.8 (21.4) | 49.9 (25.3) |
| **Annual change in ventricle volume, mL/year** | 1.2 (1.6) | 9.3 (1.8) | 3.7 (9.3) | 3.7 (5.3) | 1.1 (2.4) | 2.7 (4.0) | 4.6 (3.7) |
| **Age, years** | 45.3 (16.6) | 44.5 (11.5) | 45.7 (11.2) | NR | 76.4 (5.2) | 74.7 (7.3) | 74.9 (15.0) |

CSF1R-ALSP=colony stimulating factor 1 receptor‒adult-onset leukoencephalopathy with axonal spheroids and pigmented glia; HSCT=haematopoietic stem cell transplant; NR=not reported; SD=standard deviation.

## **Supplementary Figure 1 Location of *CSF1R* variants identified in individuals included in the study.**


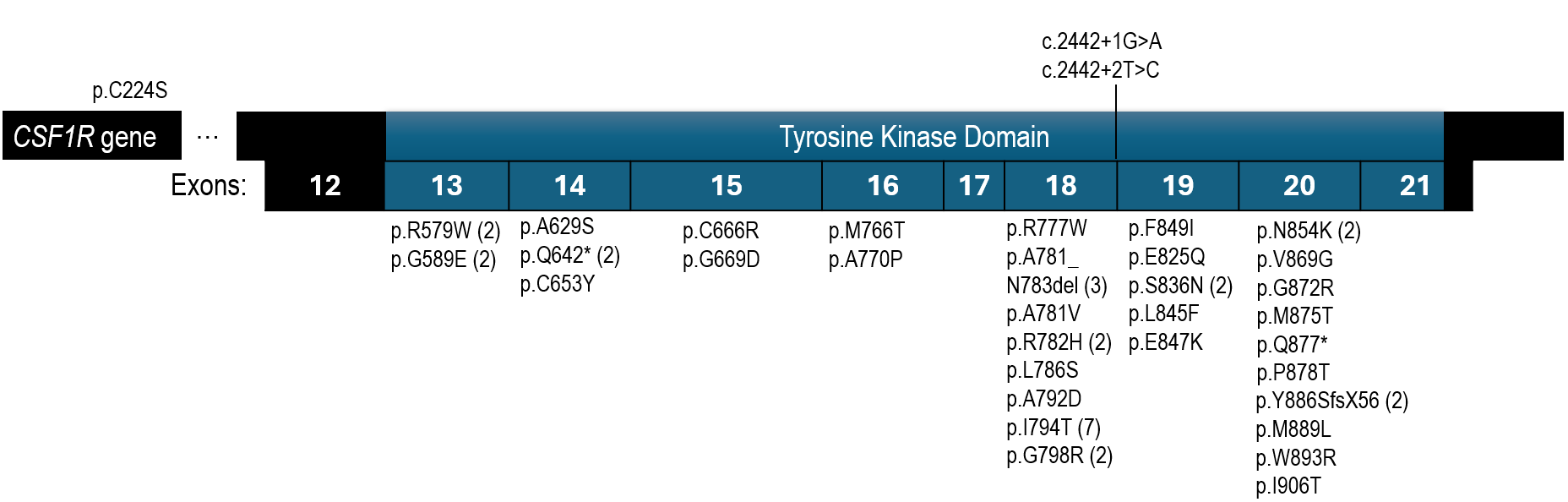


In the schematic gene representation, variants within the tyrosine kinase domain are grouped by exon. For variants found in multiple individuals, the case count is reported in parentheses. Detailed data on pathogenicity of individual variants detected in enrolled patients are summarised in Supplementary Table 3.

**Supplementary Figure 2 (A) CGI-S and (B) PGI-S assessments at baseline.**


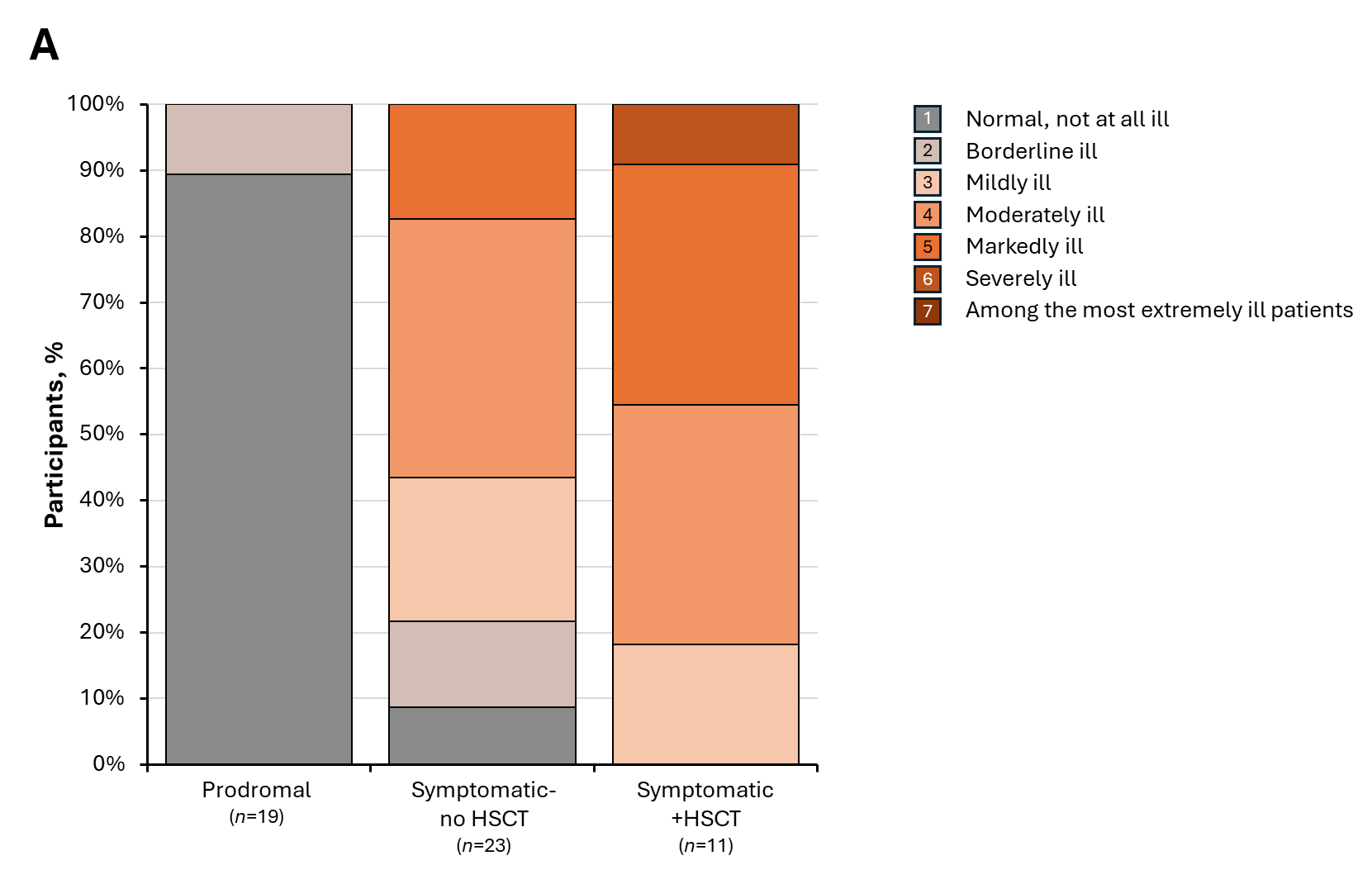


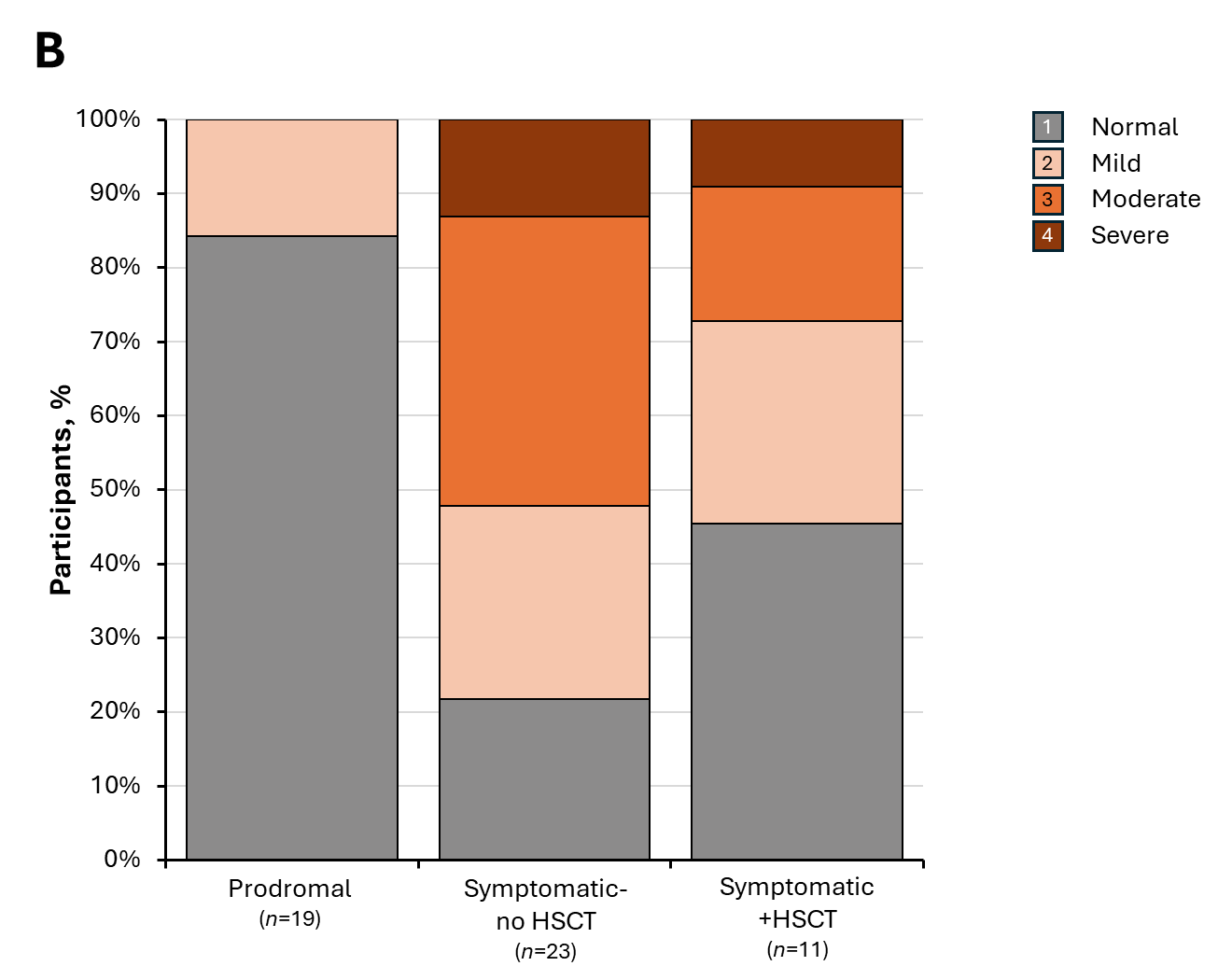


The Clinical Global Impression–Severity of Illness and Patient Global Impression–Severity of Illness scales were used to assess baseline disease severity. For the CGI-S, clinicians rated baseline severity using a seven-point scale relative to past experience with CSF1R-ALSP patients. For the PGI-S, patients rated the current severity of their disease on a four-point scale (1=normal, 2=mild, 3=moderate, 4=severe). The staked bar graphs represent the percentage of patients with each score at baseline.

CGI-S=Clinical Global Impression–Severity of Illness; CSF1R-ALSP= colony-stimulating factor 1 receptor‒related adult-onset leukoencephalopathy with axonal spheroids and pigmented glia; HSCT=haematopoietic stem cell transplant; PGI-S=Patient Global Impression–Severity of Illness.

**Supplementary Figure 3 Correlations between cognitive decline and MRI ventricle volume (A) at baseline and (B) longitudinally for changes from baseline to 12 months.**

**
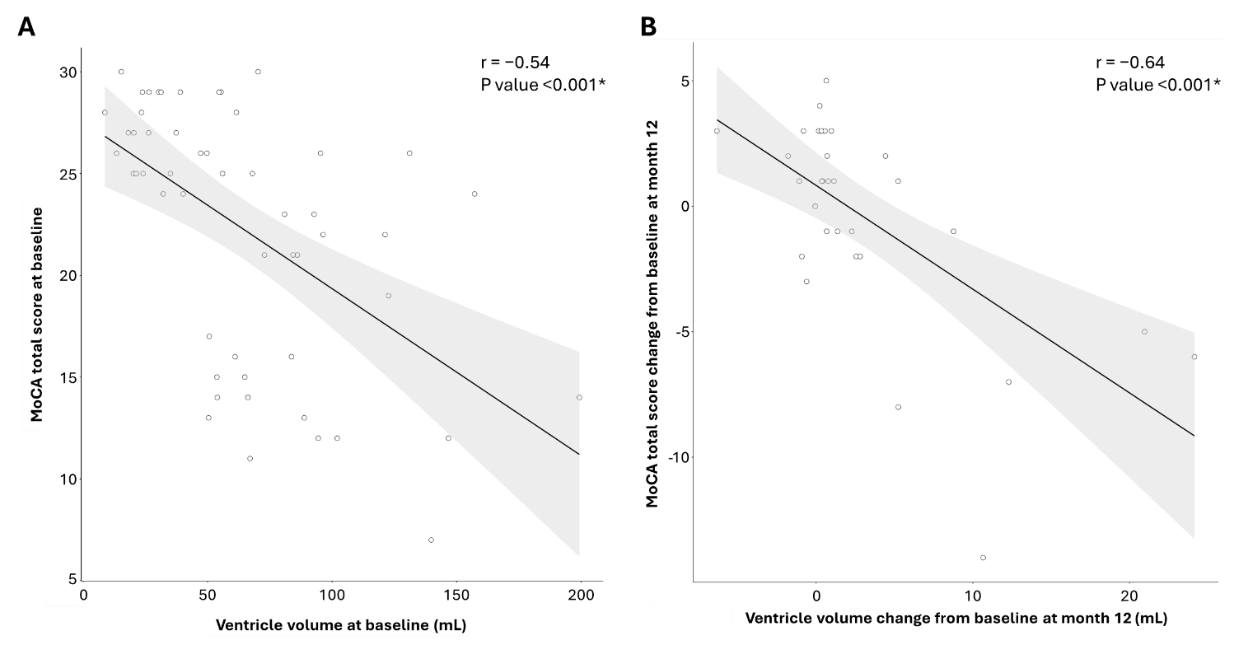
**

Includes all study participants with 12 months of available follow-up. Plotted data are individual participant values (**A**) at baseline and (**B**) for change from baseline to month 12. Pearson correlation analyses were used to assess the correlation between ventricle volume and MoCA total scores at baseline and change from baseline at month 12.

*Significant at an adjusted alpha of P<0.0009 based on Bonferroni correction.

MoCA=Montreal Cognitive Assessment; MRI=magnetic resonance imaging.

**Supplementary Figure 4 Correlations between MRI and clinical outcome assessments at baseline.**


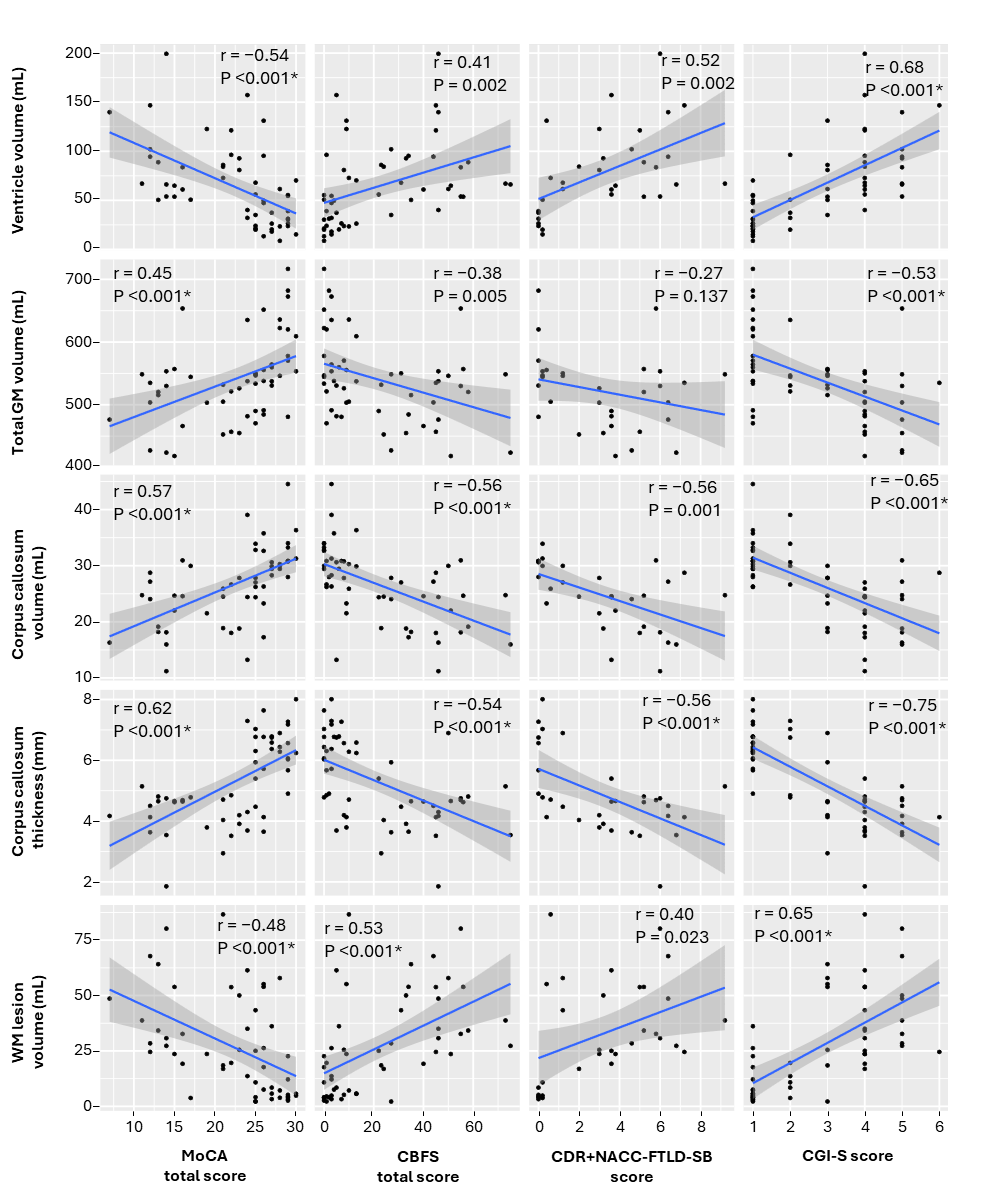


Includes all study participants with available baseline values. Plotted data are individual participant values at baseline. Pearson correlation analyses were used to assess the correlation between MRI and clinical outcome assessments at baseline.

*Significant at an adjusted alpha of P<0.0009 based on Bonferroni correction.

CBFS=Cortical Basal ganglia Functional Scale; CDR+NACC-FTLD-SB=Clinical Dementia Rating Scale plus National Alzheimer’s Coordinating Center-Frontotemporal Lobar Degeneration sum of boxes; CGI-S=Clinical Global Impression–Severity of Illness; GM=grey matter; MoCA=Montreal Cognitive Assessment; MRI=magnetic resonance imaging; WM=white matter.

**Supplementary Figure 5 Correlations between MRI and clinical outcome assessments longitudinally for changes from baseline to 12 months.**


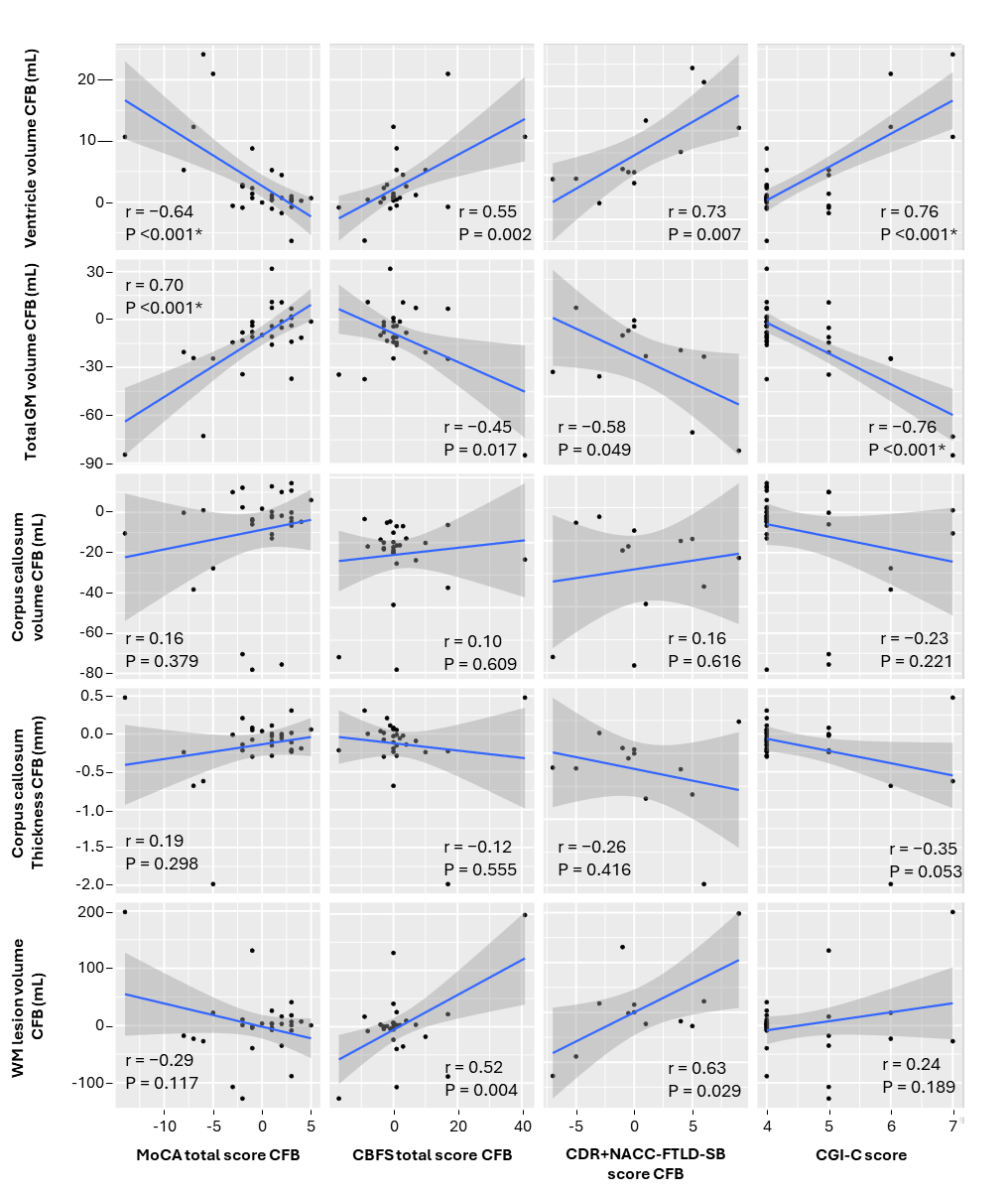


Includes all study participants with 12 months of available follow-up. Plotted data are individual participant values for change from baseline to month 12. Pearson correlation analyses were used to assess the correlation between MRI and clinical outcome assessments longitudinally for changes from baseline to 12 months.

*Significant at an adjusted alpha of P<0.0009 based on Bonferroni correction.

CBFS=Cortical Basal ganglia Functional Scale; CDR+NACC-FTLD-SB=Clinical Dementia Rating Scale plus National Alzheimer’s Coordinating Center-Frontotemporal Lobar Degeneration sum of boxes; CFB=change from baseline; CGI-C=Clinical Global Impression of Change; GM=grey matter; MoCA=Montreal Cognitive Assessment; MRI=magnetic resonance imaging; WM=white matter.

**Supplementary Figure 6 Correlations between fluid biomarkers and clinical outcome assessments at baseline.**


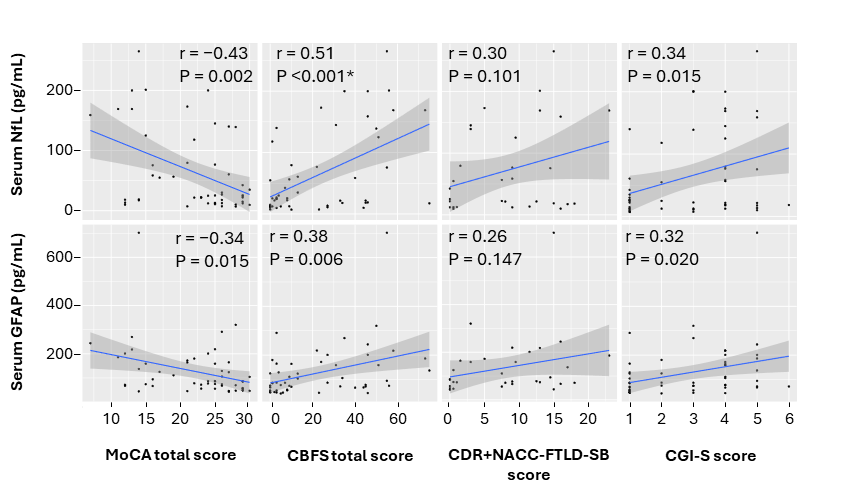

Includes all study participants with available baseline values. Plotted data are individual participant values at baseline. Pearson correlation analyses were used to assess the correlation between fluid biomarkers and clinical outcome assessments at baseline.

*Significant at an adjusted alpha of P<0.0009 based on Bonferroni correction.

CBFS=Cortical Basal ganglia Functional Scale; CDR+NACC-FTLD-SB=Clinical Dementia Rating Scale plus National Alzheimer’s Coordinating Center-Frontotemporal Lobar Degeneration sum of boxes; CGI-S=Clinical Global Impression–Severity of Illness; GFAP=glial fibrillary acidic protein; MoCA=Montreal Cognitive Assessment; NfL=neurofilament light chain.

**Supplementary Figure 7 Correlations between** **fluid biomarkers and clinical outcome assessments longitudinally for changes from baseline to 12 months.**


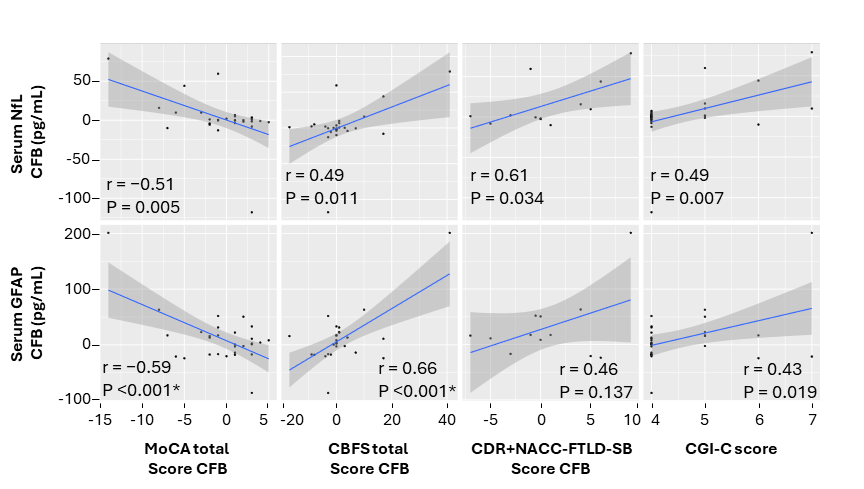


Includes all study participants with 12 months of available follow-up. Plotted data are individual participant values for change from baseline to month 12. Pearson correlation analyses were used to assess the correlation between fluid biomarkers and clinical outcome assessments longitudinally for changes from baseline to 12 months.

*Significant at an adjusted alpha of P<0.0009 based on Bonferroni correction.

CBFS=Cortical Basal ganglia Functional Scale; CDR+NACC-FTLD-SB=Clinical Dementia Rating Scale plus National Alzheimer’s Coordinating Center-Frontotemporal Lobar Degeneration sum of boxes; CFB=change from baseline; CGI-C=Clinical Global Impression of Change; GFAP=glial fibrillary acidic protein; MoCA=Montreal Cognitive Assessment; NfL=neurofilament light chain.

### **Supplementary References**

S1. Smith SM, Zhang Y, Jenkinson M, et al. Accurate, robust, and automated longitudinal and cross-sectional brain change analysis. *Neuroimage*. 2002;17(1):479-489.

S2. University of Oxford Wellcome Centre for Integrative Neuroimaging. FSL-FMRIB Software Library. Accessed November 15, 2024. [www.fmrib.ox.ac.uk/fsl](https://heliosmedicalcomms.sharepoint.com/sites/1585VIGLNaturalHistoryNHSManuscript/Shared%20Documents/01_Initial%20Submission/www.fmrib.ox.ac.uk/fsl)

S3. Jenkinson M, Bannister P, Brady M, Smith S. Improved optimization for the robust and accurate linear registration and motion correction of brain images. *Neuroimage*. 2002;17(2):825-841.

S4. Jenkinson M, Beckmann CF, Behrens TE, Woolrich MW, Smith SM. FSL. *Neuroimage*. 2012;62(2):782-790.

S5. Griffanti L, Zamboni G, Khan A, et al. BIANCA (Brain Intensity AbNormality Classification Algorithm): a new tool for automated segmentation of white matter hyperintensities. *Neuroimage*. 2016;141:191-205.

S6. Zhang Y, Brady M, Smith S. Segmentation of brain MR images through a hidden Markov random field model and the expectation-maximization algorithm. *IEEE Trans Med Imaging*. 2001;20(1):45-57.

S7. Patenaude B, Smith SM, Kennedy DN, Jenkinson M. A Bayesian model of shape and appearance for subcortical brain segmentation. *Neuroimage*. 2011;56(3):907-922.

S8. Witherspoon JW, Vasavada R, Logaraj RH, et al. Two-minute versus 6-minute walk distances during 6-minute walk test in neuromuscular disease: is the 2-minute walk test an effective alternative to a 6-minute walk test? *Eur J Paediatr Neurol*. 2019;23(1):165-170.

S9. Ibrahim A, Singh DKA, Shahar S, Omar MA. Timed up and go test combined with self-rated multifactorial questionnaire on falls risk and sociodemographic factors predicts falls among community-dwelling older adults better than the timed up and go test on its own. *J Multidiscip Healthc*. 2017;10:409-416.

S10. Pfeffer RI, Kurosaki TT, Harrah CH, Jr., Chance JM, Filos S. Measurement of functional activities in older adults in the community. *J Gerontol*. 1982;37(3):323-329.

S11. Knight BG, Fox LS, Chou CP. Factor structure of the burden interview. *J Clin Geropyschol*. 2000;6(4):249-258.

S12. Meier A, Papapetropoulos S, Marsh A, et al. Phase 1, first-in-human, single-/multiple-ascending dose study of iluzanebart in healthy volunteers. *Ann Clin Transl Neurol*. 2025;12(5):1065-1076.

S13. Genovese AV, Hagemeier J, Bergsland N, et al. Atrophied brain T2 lesion volume at MRI is associated with disability progression and conversion to secondary progressive multiple sclerosis. *Radiology*. 2019;293(2):424-433.

S14. Nestor SM, Rupsingh R, Borrie M, et al. Ventricular enlargement as a possible measure of Alzheimer's disease progression validated using the Alzheimer's disease neuroimaging initiative database. *Brain*. 2008;131(Pt 9):2443-2454.
